# The Asian Society of Endometriosis and Adenomyosis guidelines for managing adenomyosis

**DOI:** 10.1002/rmb2.12535

**Published:** 2023-09-10

**Authors:** Tasuku Harada, Fuminori Taniguchi, Sun‐Wei Guo, Young Min Choi, Kutay Omer Biberoglu, Shaw‐Jenq Sean Tsai, Saeed Alborzi, Moamar Al‐Jefout, Amphan Chalermchokcharoenkit, Angela G. Sison‐Aguilar, Yoke‐Fai Fong, Hemantha Senanayake, Alexander Popov, Andon Hestiantoro, Yuval Kaufman

**Affiliations:** ^1^ Faculty of Medicine, Tottori University Yonago Japan; ^2^ OB/GYN Hospital Fudan University Shanghai China; ^3^ Seoul National University College of Medicine Seoul Korea; ^4^ Gazi University Medical School Turkey Turkey; ^5^ College of Medicine National Cheng Kung University Tainan City Taiwan; ^6^ Shiraz University of Medical Sciences Shiraz Iran; ^7^ United Arab Emirates University, College of Medicine and Health Sciences Abu Dhabi UAE; ^8^ Siriraj Hospital, Mahidol University Bangkok Thailand; ^9^ University of the Philippines College of Medicine Manila Philippines; ^10^ National University of Singapore Singapore City Singapore; ^11^ The University of Colombo Colombo Sri Lanka; ^12^ Moscow Regional Scientific Research Institute of Obstetrics and Gynecology Moscow Russia; ^13^ University of Indonesia Jakarta Indonesia; ^14^ Carmel Medical Center Haifa Israel

## Abstract

This is the first guidelines for adenomyosis from the Asian Society of Endometriosis and Adenomyosis.

**Table:** 

Table of Contents
1. INTRODUCTION
2. OVERVIEW
2‐1. Diagnosis
2‐2. Medical treatment
2‐3. Surgical treatment
CLINICAL QUESTIONS (CQS)
CQ1: Is imaging useful for the diagnosis of adenomyosis?
CQ2: Are hormonal agents effective for adenomyosis‐associated pain?
CQ3: Is surgical removal effective for adenomyosis‐associated pain?
CQ4: Are non‐nonsurgical treatments effective for adenomyosis‐associated pain?
CQ5: Is surgical removal effective for adenomyosis‐associated infertility?
CQ6: Are hormonal agents effective for adenomyosis‐associated infertility?
CQ7: Does adenomyosis influence pregnancy complications?
CQ8: Does the removal of adenomyosis lesions influence pregnancy complications?
CQ 9: Does hormonal treatment of adenomyosis influence the pregnancy outcome?

## 1 INTRODUCTION

First described nearly two centuries ago, adenomyosis remains to this day a huge clinical challenge due to its complex symptomology, enigmatic pathogenesis, and pathophysiology. In addition, its prevalence appears to vary among different racial and ethnic groups as well as geographic regions.^1^ For reasons that are still unknown, Asian women are more likely to be diagnosed with adenomyosis as compared to their Caucasian counterparts (OR = 1.99, 95% confidence interval (CI) = 1.19–3.32).^1^, ^2^

As endometriosis had been increasingly recognized as one of the major health issues of women considering its physical and psychological impact, numerous national, regional, and international guidelines on managing this entity had been established across the world. Nonetheless, despite the prevalence of adenomyosis and its negative impact—certainly no smaller than that of endometriosis—on women's quality of life and the challenge in its management, so far there has not been a widely accepted or even published guideline on the management of adenomyosis. The burden of adenomyosis on patients and the health system had been proposed to be substantial considering its clinical symptoms and the cost of its management it incurs.^3^ Furthermore, the negative impact of adenomyosis on reproductive outcomes in terms of decreased pregnancy rates and increased risk of miscarriage had been reported.^4^ Even though the awareness of adenomyosis has increased in recent two decades as evidenced by the growing number of publications, a lack of a regional or international guideline reflects, perhaps in no small part, the enormous heterogeneity in diagnosis and treatment, as seen, for example, from substantial differences between criteria for the histopathologic study and imaging study^5^ and the fact that as of now well over 95% of fertility‐sparing adenomyomectomy surgeries worldwide are performed in Asia. In addition, there exist several classification methods of adenomyosis based on morphology.^6^ The establishment of a widely accepted approach to the diagnosis and classification of adenomyosis certainly needs extensive discussion.

Due mostly to poorly understood pathophysiology of adenomyosis, its management still poses a great challenge today.^7^ To manage adenomyosis and its negative sequela, a multidisciplinary approach is often required, and fertility must be considered. Depending on the patient's age, reproductive status, clinical symptoms and their severity, and desires and wishes for pregnancy, adenomyosis requires individualized and comprehensive treatment.^8^ Several nonsurgical and minimally invasive, fertility‐sparing surgical treatment options have been developed and the management plan ought to be individualized to meet the patient's expectations.^9^ However, the management of adenomyosis for women with ongoing reproductive needs shall be necessarily based on accumulating evidence on the efficacy of a particular strategy and its fertility outcomes to guide clinical practice. Therefore, the establishment of a consensus on the management of adenomyosis would be beneficial in providing comprehensive and appropriate care for affected women.

The establishment of guidelines for adenomyosis is necessary to help clinicians make the best possible decision during care for affected women based on the evidence as much as possible. It should also expose gray and dark areas of knowledge that are in need for more research. Asia is the most populated continent in the world and presumably has the highest number of affected women. It is also the continent that first adopted dienogest to treat adenomyosis and witnessed the advent of adenomyomecomy operations in the world. Therefore, as the only professional society on adenomyosis in the continent, we think that it is high time to establish a guideline on the management of adenomyosis and take upon ourselves this task.

REFERENCES1

Bougie
O
, 
Yap
MI
, 
Sikora
L
, 
Flaxman
T
, 
Singh
S
. Influence of race/ethnicity on prevalence and presentation of endometriosis: a systematic review and meta‐analysis. BJOG.
2019;126(9):1104–1115. 10.1111/1471-0528.15692
309088742

Naftalin
J
, 
Hoo
W
, 
Pateman
K
, 
Mavrelos
D
, 
Holland
T
, 
Jurkovic
D
. How common is adenomyosis? A prospective study of prevalence using transvaginal ultrasound in a gynaecology clinic. Hum Reprod.
2012;27(12):3432–3439. 10.1093/humrep/des332
230017753

Yu
O
, 
Schulze‐Rath
R
, 
Grafton
J
, 
Hansen
K
, 
Scholes
D
, 
Reed
SD
. Adenomyosis incidence, prevalence and treatment: United States population‐based study 2006‐2015. Am J Obstet Gynecol. 2020;223:94.e1–94.e10. 10.1016/j.ajog.2020.01.016
319541564

Harada
T
, 
Khine
YM
, 
Kaponis
A
, 
Nikellis
T
, 
Decavalas
G
, 
Taniguchi
F
. The impact of adenomyosis on women's fertility. Obstet Gynecol Surv. 2016;71(9):557–568. 10.1097/OGX.0000000000000346
27640610PMC50499765

Habiba
M
, 
Benagiano
G
. Classifying adenomyosis: progress and challenges. Int J Environ Res Public Health. 2021;18(23):12386. 10.3390/ijerph182312386
34886111PMC86565146

Guo
SW
, 
Benagiano
G
, 
Bazot
M
. In search of an imaging classification of adenomyosis: a role for elastography. J Clin Med.
2022;12(1):287. 10.3390/jcm12010287
36615089PMC98211567

Chapron
C
, 
Vannuccini
S
, 
Santulli
P
, 
Abrao
MS
, 
Carmona
F
, 
Fraser
IS
,et al. Diagnosing adenomyosis: an integrated clinical and imaging approach. Hum Reprod Update. 2020;26(3):392–411. 10.1093/humupd/dmz049
320974568

Sharara
FI
, 
Kheil
MH
, 
Feki
A
, 
Rahman
S
, 
Klebanoff
JS
, 
Ayoubi
JM
, et al. Current and prospective treatment of adenomyosis. J Clin Med.
2021;10(15):3410. 10.3390/jcm10153410
34362193PMC83481359

Vannuccini
S
, 
Luisi
S
, 
Tosti
C
, 
Sorbi
F
, 
Petraglia
F
. Role of medical therapy in the management of uterine adenomyosis. Fertil Steril.
2018;109(3):398–405. 10.1016/j.fertnstert.2018.01.013
29566852

## 2 OVERVIEW

### 2.1 Diagnosis

Adenomyosis is associated with dysmenorrhea, abnormal uterine bleeding manifesting mostly as heavy menstrual bleeding (HMB), pelvic pain, dyspareunia, and reduced fertility, although about one‐third of patients are reported to be asymptomatic.^1^ However, these symptoms are unfortunately not specific to adenomyosis, and, as such, the diagnosis must rely on other means.^2^ As in almost all diseases, the diagnosis process of adenomyosis typically starts with history taking, evaluation of basic demographic and reproductive information such as age, age at menarche, gravidity, and parity, the clinical presentation of symptoms and signs, family history, and the quality of life, leading to suspicion of the disease.

When adenomyosis is suspected, a bimanual examination of the pelvis can help physicians to gauge the uterine size and mobility, and adnexal masses, followed by the assessment of pelvic pain, and, if so, type, severity, and localization of pain, to raise or rule out the possibility of the presence of deep endometriosis in the retrocervical region. Next, noninvasive imaging examination should be employed.

In the last three decades, magnetic resonance imaging (MRI) and ultrasound have gradually become the mainstay for the diagnosis of adenomyosis, completely replacing histological evaluation following hysterectomy as the main diagnostic tool. In particular, transvaginal ultrasound (TVUS) is the first‐line technique in gynecological work‐up because it is widely available, easier than MRI to operate but less expensive than MRI, and also allows a dynamic examination to explore organ mobility and site‐specific tenderness. Through two‐dimensional (2D) and 3D settings and color flow Doppler versions of TVUS, a good view of the uterus and its pathology can be obtained. Compared with TVUS, transabdominal ultrasonography has limited value but can be a good alternative when the vaginal route is inaccessible or in case of a grossly enlarged uterus.^3^ Compared with 2D and 3D TVUS, color flow Doppler ultrasonography has the added advantage of providing information on the location, amount, and type of blood flow.^4^, ^5^ This can help to differentiate adenomyosis from uterine fibroids (UFs), enhancing the overall diagnostic accuracy.^6^ In the hands of a trained sonographer, TVUS is also quite very accurate in diagnosing other gynecological pathologies, such as ovarian endometrioma.

In diagnosing adenomyosis by TVUS, several ultrasonographic features have been proposed. These include uterine enlargement, asymmetry of anterior and posterior uterine walls, heterogeneous myometrium, presence of myometrial cysts, heterogeneous myometrium, hyperechoic or hypoechoic linear striation in the myometrium, poorly delineated junctional zone (JZ), the presence of echogenic striations in the sub‐endometrium, subendometrial echogenic nodules.^7^, ^8^, ^9^, ^10^, ^11^

A recent meta‐analysis of all diagnostic performance studies published during 2015–2020 yielded a combined sensitivity and specificity of 0.82 (95% CI = 0.77–0.86) and 0.81 (95% CI = 0.66–0.90), respectively, for TVUS.^12^ However, its diagnostic accuracy can be compromised substantially when UF is also present because of the circumscribed nature.^13^, ^14^

MRI is also useful in identifying the location, number, and extent of adenomyotic lesions. Like 3D TVUS, the zonal anatomy of the uterus can be demonstrated on T2‐weighted images. Even to untrained eyes, it provides clear pictures of the pelvic anatomy and the uterus, in the either sagittal, coronal, or transverse plane, and in a slice‐by‐slice manner. Because of its limited availability and higher cost, however, it is often employed in a second‐line work‐up, especially after inconclusive TVUS investigation.^13^ Compared with TVUS, MRI provides more detailed intrapelvic information, allowing concurrent diagnosis of ovarian endometrioma and deep endometriosis. In addition, it has superior objectivity when diagnosing adenomyosis.

The sensitivity and specificity of MRI for diagnosing adenomyosis range from 88%–93% and 67%–91%, respectively,^15^ nearly equal to that of TVUS.^14^, ^16^, ^17^ However, it is less operator dependent, more subjective, and relies less on the capacity of the observer to diagnose. Indeed, the substantially heterogeneous diagnostic criteria certainly do not enhance the edge of TVUS.^7^, ^16^

To overcome this heterogeneity, an international expert panel published in 2015 the Morphological Uterus Sonographic Assessment (MUSA) consensus statement on the descriptive markers for the diagnosis of adenomyosis on TVUS, to provide a standardized terminology for describing ultrasound images of normal and pathological myometrium.^18^ The same group also published a consensus on the standardized classification and reporting of adenomyosis based on TVUS.^19^ Important items that should be reported include lesion location, the distinction between focal and diffuse adenomyosis, identification of cystic/noncystic elements, and involvement of myometrial layer, which is grouped into three types: inner/sub‐endometrial myometrium (Type I), middle myometrium (Type II) and outer/sub‐serosal myometrium (Type III). In addition, the disease extension is classified as mild, moderate, or severe, and the measurement of lesion size.^19^

While these efforts undoubtedly help improve the diagnostic accuracy of TVUS, few have ever questioned whether the current TVUS instrumentation/technology may have reached its physical limit. After all, there are limits to human discovery, and there are ultimately unknowable, undoable, or unreachable.^20^ This happened in diagnosing deep endometriosis when lesions are small.^21^

Raising this question and confronting it honestly can be sobering and helpful, since this would prompt us to think of possible solutions and other options, such as sonohysterography, hysteroscopy, and elastography.^15^

REFERENCES1

Bergeron
C
, 
Amant
F
, 
Ferenczy
A
. Pathology and physiopathology of adenomyosis. Best Pract Res Clin Obstet Gynaecol.
2006;20(4):511–521.1656387010.1016/j.bpobgyn.2006.01.0162

Peric
H
, 
Fraser
IS
. The symptomatology of adenomyosis. Best Pract Res Clin Obstet Gynaecol. 2006;20:547–555. 10.1016/j.bpobgyn.2006.01.006
165158883

Bazot
M
, 
Darai
E
, 
Rouger
J
, 
Detchev
R
, 
Cortez
A
, 
Uzan
S
. Limitations of transvaginal sonography for the diagnosis of adenomyosis, with histopathological correlation. Ultrasound Obstet Gynecol. 2002;20:605–611. 10.1046/j.1469-0705.2002.00852.x
124930514

Valentini
AL
, 
Speca
S
, 
Gui
B
, 
Soglia
G
, 
Micco
M
, 
Bonomo
L
. Adenomyosis: from the sign to the diagnosis. Imaging, diagnostic pitfalls, and differential diagnosis: a pictorial review. Radiol Med. 2011;116:1267–1287. 10.1007/s11547-011-0714-5
218927205

Exacoustos
C
, 
Manganaro
L
, 
Zupi
E
. Imaging for the evaluation of endometriosis and adenomyosis. Best Pract Res Clin Obstet Gynaecol. 2014;28:655–681. 10.1016/j.bpobgyn.2014.04.010
248612476

Andres
MP
, 
Borrelli
GM
, 
Ribeiro
J
, 
Baracat
EC
, 
Abrao
MS
, 
Kho
RM
. Transvaginal ultrasound for the diagnosis of adenomyosis: systematic review and meta‐analysis. J Minim Invasive Gynecol. 2018;25:257–264. 10.1016/j.jmig.2017.08.653
288640447

Dartmouth
K
. A systematic review with meta‐analysis: the common sonographic characteristics of adenomyosis. Ultrasound. 2014;22:148–157. 10.1177/1742271X14528837
27433212PMC47605308

Kepkep
K
, 
Tuncay
YA
, 
Goynumer
G
, 
Tutal
E
. Transvaginal sonography in the diagnosis of adenomyosis: which findings are most accurate?
Ultrasound Obstet Gynecol. 2007;30:341–345. 10.1002/uog.3985
176596499

Levy
G
, 
Dehaene
A
, 
Laurent
N
, 
Lernout
M
, 
Collinet
P
, 
Lucot
JP
, et al. An update on adenomyosis. Diagn Interv Imaging. 2013;94:3–25. 10.1016/j.diii.2012.10.012
2324618610

Graziano
A
, 
Lo Monte
G
, 
Piva
I
, 
Caserta
D
, 
Karner
M
, 
Engl
B
, et al. Diagnostic findings in adenomyosis: a pictorial review on the major concerns. Eur Rev Med Pharmacol Sci. 2015;19:1146–1154.2591257211

Shwayder
J
, 
Sakhel
K
. Imaging for uterine myomas and adenomyosis. J Minim Invasive Gynecol. 2014;21:362–376. 10.1016/j.jmig.2013.11.011
2431613812

Liu
L
, 
Li
W
, 
Leonardi
M
, 
Condous
G
, 
Da Silva
CF
, 
Mol
BW
, et al. Diagnostic accuracy of transvaginal ultrasound and magnetic resonance imaging for adenomyosis: systematic review and meta‐analysis and review of sonographic diagnostic criteria. J Ultrasound Med. 2021;40:2289–2306. 10.1002/jum.15635
3350276713

Tamai
K
, 
Koyama
T
, 
Umeoka
S
, 
Saga
T
, 
Fujii
S
, 
Togashi
K
. Spectrum of MR features in adenomyosis. Best Pract Res Clin Obstet Gynaecol. 2006;20:583–602. 10.1016/j.bpobgyn.2006.01.009
1656422814

Dueholm
M
, 
Lundorf
E
, 
Hansen
ES
, 
Sorensen
JS
, 
Ledertoug
S
, 
Olesen
F
. Magnetic resonance imaging and transvaginal ultrasonography for the diagnosis of adenomyosis. Fertil Steril. 2001;76:588–594. 10.1016/s0015-0282(01)01962-8
1153248615

Chapron
C
, 
Vannuccini
S
, 
Santulli
P
, 
Abrao
MS
, 
Carmona
F
, 
Fraser
IS
, et al. Diagnosing adenomyosis: an integrated clinical and imaging approach. Hum Reprod Update. 2020;26:392–411. 10.1093/humupd/dmz049
3209745616

Bazot
M
, 
Darai
E
. Role of transvaginal sonography and magnetic resonance imaging in the diagnosis of uterine adenomyosis. Fertil Steril. 2018;109:389–397. 10.1016/j.fertnstert.2018.01.024
2956685117

Bazot
M
, 
Cortez
A
, 
Darai
E
, 
Rouger
J
, 
Chopier
J
, 
Antoine
JM
, et al. Ultrasonography compared with magnetic resonance imaging for the diagnosis of adenomyosis: correlation with histopathology. Hum Reprod. 2001;16:2427–2433. 10.1093/humrep/16.11.2427
1167953318

Van den Bosch
T
, 
Dueholm
M
, 
Leone
FP
, 
Valentin
L
, 
Rasmussen
CK
, 
Votino
A
, et al. Terms, definitions, and measurements to describe sonographic features of myometrium and uterine masses: a consensus opinion from the Morphological Uterus Sonographic Assessment (MUSA) group. Ultrasound Obstet Gynecol. 2015;46:284–298. 10.1002/uog.14806
2565268519

Van den Bosch
T
, 
de Bruijn
AM
, 
de Leeuw
RA
, 
Dueholm
M
, 
Exacoustos
C
, 
Valentin
L
, et al. Sonographic classification and reporting system for diagnosing adenomyosis. Ultrasound Obstet Gynecol. 2019;53:576–582. 10.1002/uog.19096
2979021720

Barrow
JD
. Impossibility: the limits of science and the science of limits. New York: Oxford Univesity Press; 1999. p. 304.21

Ding
D
, 
Chen
Y
, 
Liu
X
, 
Jiang
Z
, 
Cai
X
, 
Guo
SW
. Diagnosing deep endometriosis using transvaginal elastosonography. Reprod Sci. 2020;27:1411–1422. 10.1007/s43032-019-00108-2
32333226

### 2.2 Medical treatment

Dysmenorrhea and HMB are the top two symptoms of adenomyosis, presenting in 50%–93% and 27%–65% of patients, respectively.^1^ The other clinical symptoms include dyspareunia, chronic pelvic pain, and infertility, although about one‐third of the affected patients are asymptomatic.^2^, ^3^ The lack of information about the nature and pathophysiology of adenomyosis and the paucity of a proper universal classification system has led to a near anarchy in the treatment of this disease.^4^, ^5^, ^6^ Due, perhaps in no small part, to the lack of a guideline that prioritizes one treatment over another in this field.^7^

Like endometriosis, adenomyosis is an estrogen‐responsive condition, which forms the basis of medical treatment by controlling the hormone milieu.^8^ Medical treatment of adenomyosis is always the first line of treatment, but it can be used if the patient does not intend to get pregnant. Additionally, it should be noted that the relapse of the disease is inevitable after cessation of the medical treatment.^9^ Therefore, the indication of nonsurgical medical treatment appears in patients with adenomyosis who desire to preserve their uteri has the intention to get pregnant, patients near menopause, or those who are contraindicated for surgical treatment due to other medical comorbidities.^10^ The proposed medical treatments for adenomyosis include combined oral contraceptive (COC) pills, progesterone pills, LNG‐IUS, GnRH agonists (GnRHa), GnRH antagonists, dienogest, danazol, as well as some experimental drugs, such as aromatase inhibitors, antiplatelet drugs, oxytocin antagonist medications. In the literature review, there are few published randomized clinical trials on the effectiveness of different drug options on adenomyosis, especially based on the head‐to‐head comparison. Very often, published trials have a short follow‐up period of just 4–12 weeks.^11^, ^12^, ^13^, ^14^, ^15^

Accordingly, with COCs, GnRHa, and aromatase inhibitor medications, the two main symptoms of adenomyosis, namely HMB and chronic pelvic pain, can be relieved and improved. However, in terms of reducing the uterine volume and improving the quality of life in the affected patients, information is typically scanty except for the studies conducted on LNG‐IUS with a follow‐up period of about 12 months. More conclusive studies with a long follow‐up time are needed so that a definitive decision could be made.^10^

By inhibiting FSH and LH and thus estrogen biosynthesis, the COCs pills can suppress the growth of the follicles and the progress of endometrial proliferation. Moreover, by inhibiting the menstrual cycle, they can induce a state of amenorrhea and thus control the symptoms related to menstruation.^16^ There are, however, no data on their effectiveness on adenomyotic lesions themselves, and most of the findings are based on the tissue responses to treatment in conditions where adenomyosis coexists with endometriosis or leiomyoma.^17^, ^18^, ^19^

One study reported that intrinsic adenomyosis, that is, lesions that are confined to regions in proximity to the endometrium, is more sensitive to the spotting, as a side effect, when treated with progesterone.^20^ Nonetheless, research on the relationship between types of adenomyosis and the specific side effects is quite limited.^21^

Of course, the issue of progesterone resistance should not be ignored, which causes refraction to treatment in about one‐third of cases due most likely to the inactivation of the progesterone receptor, the lack of enough receptors in the target tissue,^22^, ^23^ or KRAS mutation.^24^

In the meantime, dienogest, which is a relatively new selective synthetic oral progestin, has good effects on endometriosis‐associated pain and is being used for the treatment of adenomyosis symptoms. In several studies, the effect of dienogest on relieving dysmenorrhea is equal or superior to that of COCs and GnRHa drugs, but in terms of reducing the uterine volume and induction of amenorrhea, it is less effective than those drugs while more side effects have been reported. Further research with longer follow‐up is needed in this area.^25^, ^26^, ^27^, ^28^, ^29^

LNG‐IUS can improve all the symptoms associated with adenomyosis by inducing decidualization and atrophy of the endometrium and downregulation of estrogen receptors by increasing the release of progesterone.^30^, ^31^ Today, this option is used worldwide as the first‐line medical treatment for adenomyosis, which reduces pain, uterine volume, as well as HMB, seemingly more efficacious than COCs. It is also described as a highly effective option for the treatment of adenomyosis. The National Institute for Health and Care Excellence (NICE) of the United Kingdom strongly recommends the use of LNG‐IUS for the treatment of adenomyosis.^32^, ^33^, ^34^

Conceivably, adenomyosis‐caused infertility is attributable to elevated local estrogen levels and chronic inflammation in the endometrial environment, resulting in uterine hypercontractibility or dysperistalsis, impairment of sperm transport in the female genital tract, disruption of endometrial stromal decidualization, and progesterone resistance. In the meantime, attention has been paid to fertility improvement and the outcome of pregnancy with GnRHa medications; with the antiproliferative effects of these medications on the myometrium and the reduction they induce in the level of estradiol, they reduce the uterine size and result in amenorrhea and improved adenomyosis‐related pain symptoms after 6 months of treatment.^35^, ^36^

However, the use of GnRHa is not recommended for the long‐term due to its hypoestrogenic effects, especially its bone loss effect; thus, its use should better be restricted to patients who have not responded to other medications or for whom surgery is not a viable option. The use of GnRHa as a pretreatment in infertile patients and before embryo transfer has received a great deal of attention.^37^, ^38^

In the last 4–5 years, the use of GnRH antagonists (GnRHant) to treat adenomyosis has been demonstrated to be very promising. While the mechanism of action for GnRHant is very similar to that of GnRHa, the ability to elimination of the “flare‐up” phenomenon and the potential to titrate dosage individually (when orally administrated) give the former a clear edge over the latter. In addition, with the option of add‐back medication, the GnRHant offers hope for long‐term usage. Preliminary data have shown that they are effective in reducing the size of the uterus and the clinical symptoms associated with adenomyosis.^39^, ^40^, ^41^, ^42^ However, its downside is also conspicuous: as of now, it is quite expensive, apparently out of reach from most patients in low‐ or even middle‐income countries. In addition, trial results appear to indicate that there is a great deal of interindividual variation in the response to the GnRHant treatment: some patients may experience amenorrhea while others may become pregnant even though the same dosage is used. How to titrate the dosage individually is still an open question. Moreover, the cost‐benefit analysis in comparison with GnRHa or even dienogest treatment has not been performed. While it offers hope for long‐term use, the possibility of malignant transformation has not been adequately assessed so far, both in the eutopic and ectopic endometrium. This is particularly concerning given the recent report that many adenomyotic lesions harbor cancer‐driver mutations such as KRAS24.

More investigation is needed regarding experimental drugs, such as bromocriptine (a dopamine agonist), an aromatase inhibitor, antiplatelet medications, and oxytocin antagonist drugs along with their effects on adenomyosis symptoms.^43^, ^44^, ^45^, ^46^

Finally, to choose the optimal medical treatment, it is necessary to act individually and pay attention to the following characteristics:

- Patient's age;
- Patient's symptoms and severity: AUB/HMB, chronic pelvic pain, hemoglobin drop;
- Desire for future fertility;
- The presence of concomitant diseases, such as endometriosis, leiomyoma, and pelvic congestion disease;
- Side effects of chosen medication;
- Cost and availability;
- Patient's wishes.

In the following, we will examine the medical therapeutic strategies based on the available evidence, and by responding to the clinical questions, a suitable guideline for the medical treatment of adenomyosis will be provided based on the approach to pain and infertility and improving the outcome of pregnancy.

REFERENCES1

Bergeron
C
, 
Amant
F
, 
Ferenczy
A
. Pathology and physiopathology of adenomyosis. Best Pract Res Clin Obstet Gynaecol.
2006;20(4):511–521.1656387010.1016/j.bpobgyn.2006.01.0162

Alborzi
S
, 
Askary
E
, 
Khorami
F
, 
Poordast
T
, 
Abdulwahid Hashim Alkhalidi
B
, 
Hamedi
M
, et al. A detailed study in adenomyosis and endometriosis: evaluation of the rate of coexistence between uterine adenomyosis and DIE according to imaging and histopathology findings. Reprod Sci.
2021;28(8):2387–2397. 10.1007/s43032-021-00527-0
337253133

Gordts
S
, 
Grimbizis
G
, 
Campo
R
. Symptoms and classification of uterine adenomyosis, including the place of hysteroscopy in diagnosis. Fertil Steril. 2018;109(03):380–388.e1. 10.1016/j.fertnstert.2018.01.006
295668504

Garry
R
. The endometriosis syndromes: a clinical classification in the presence of aetiological confusion and therapeutic anarchy. Hum Reprod. 2004;19:760–768.1503394410.1093/humrep/deh1475

Habiba
M
, 
Gordts
S
, 
Bazot
M
, 
Brosens
I
, 
Benagiano
G
. Exploring the challenges for a new classification of adenomyosis. Reprod Biomed Online. 2020;40:569–581.3217323910.1016/j.rbmo.2020.01.0176

Yu
O
, 
Schulze‐Rath
R
, 
Grafton
J
, 
Hansen
K
, 
Scholes
D
, 
Reed
SD
. Adenomyosis incidence, prevalence and treatment. United States population‐based study 2006–2015. Am J Obstet Gynecol. 2020;223:94.e1–94.e10.10.1016/j.ajog.2020.01.016319541567

Vannuccini
S
, 
Luisi
S
, 
Tosti
C
, 
Sorbi
F
, 
Petraglia
F
. Role of medical therapy in the management of uterine adenomyosis. Fertil Steril. 2018;109:398–405.2956685210.1016/j.fertnstert.2018.01.0138

Kitawaki
J
. Adenomyosis: the pathophysiology of an oestrogen‐dependent disease. Best Pract Res Clin Obstet Gynaecol. 2006;20:493–502.1656422710.1016/j.bpobgyn.2006.01.0109

Wong
F
, 
Zhu
XG
, 
Xue
M
. Adenomyosis – is a new treatment solution available?
Clin Exp Obstet Gynecol. 2021;48(1):5–8.10

Benetti‐Pinto
CL
, 
Mira
TAA
, 
Yela
DA
, 
Teatin‐Juliato
CR
, 
Brito
LGO
. Pharmacological treatment for symptomatic adenomyosis: a systematic review. Rev Bras Ginecol Obstet.
2019;41(9):564–574. 10.1055/s-0039-1695737
3154627811

Ozdegirmenci
O
, 
Kayikcioglu
F
, 
Akgul
MA
, 
Kaplan
M
, 
Karcaaltincaba
M
, 
Haberal
A
, et al. Comparison of levonorgestrel intrauterine system versus hysterectomy on efficacy and quality of life in patients with adenomyosis. Fertil Steril. 2011;95(02):497–502. 10.1016/j.fertnstert.2010.10.009
2107415012

Badawy
AM
, 
Elnashar
AM
, 
Mosbah
AA
. Aromatase inhibitors or gonadotropin‐releasing hormone agonists for the management of uterine adenomyosis: a randomized controlled trial. Acta Obstet Gynecol Scand. 2012;91(04):489–495. 10.1111/j.1600-0412.2012.01350.x
2222925613

Shaaban
OM
, 
Ali
MK
, 
Sabra
AM
, 
Abd El Aal
DE
. Levonorgestrel‐releasing intrauterine system versus a low‐dose combined oral contraceptive for treatment of adenomyotic uteri: a randomized clinical trial. Contraception. 2015;92(04):301–307. 10.1016/j.contraception.2015.05.015
2607167314

Fawzy
M
, 
Mesbah
Y
. Comparison of dienogest versus triptorelin acetate in premenopausal women with adenomyosis: a prospective clinical trial. Arch Gynecol Obstet. 2015;292(06):1267–1271. 10.1007/s00404-015-3755-5
2599048015

Osuga
Y
, 
Fujimoto‐Okabe
H
, 
Hagino
A
. Evaluation of the efficacy and safety of dienogest in the treatment of painful symptoms in patients with adenomyosis: a randomized, double‐blind, multicenter, placebo‐controlled study. Fertil Steril. 2017;108(04):673–678. 10.1016/j.fertnstert.2017.07.021
2891193416

Valentini
AL
, 
Speca
S
, 
Gui
B
, 
Soglia
G
, 
Micco
M
, 
Bonomo
L
. Adenomyosis: from the sign to the diagnosis. Imaging, diagnostic pitfalls and differential diagnosis: a pictorial review. Radiol Med (Torino). 2011;116(8):1267–1287. 10.1007/s11547-011-0714-5
2189272017

Sharafi
K
, 
Kunze
K
, 
Nosher
JL
, 
Bachmann
GA
. Symptomatic fibroids: the need to include low dose OCPs as a treatment option. Prim Care Update OB GYNS. 2000;7:46–48.18

Pontis
A
, 
D’Alterio
MN
, 
Pirarba
S
, 
De Angelis
C
, 
Tinelli
R
, 
Angioni
S
. Adenomyosis: a systematic review of medical treatment. Gynecol Endocrinol. 2016;32:696–700.2737997210.1080/09513590.2016.119720019

Mansouri
R
, 
Santos
XM
, 
Bercaw‐Pratt
JL
, 
Dietrich
JE
. Regression of adenomyosis on magnetic resonance imaging after a course of hormonal suppression in adolescents: a case series. J Pediatr Adolesc Gynecol. 2015;28:437–440.2623328810.1016/j.jpag.2014.12.00920

Matsubara
S
, 
Kawaguchi
R
, 
Akinishi
M
, 
Nagayasu
M
, 
Iwai
K
, 
Niiro
E
, et al. Subtype I (intrinsic) adenomyosis is an independent risk factor for dienogest‐related serious unpredictable bleeding in patients with symptomatic adenomyosis. Sci Rep. 2019;9(1):17654.
10.1038/s41598-019-54096-z
31776404PMC688134421

Kishi
Y
, 
Suginami
H
, 
Kuramori
R
, 
Yabuta
M
, 
Suginami
R
, 
Taniguchi
F
. Four subtypes of adenomyosis assessed by magnetic resonance imaging and their specification. Am J Obstet Gynecol. 2012;207:111–117.10.1016/j.ajog.2012.06.0272284071922

Vercellini
P
, 
Buggio
L
, 
Berlanda
N
, 
Barbara
G
, 
Somigliana
E
, 
Bosari
S
. Estrogen‐progestins and progestins for the management of endometriosis. Fertil Steril. 2016;106:1552.2781783710.1016/j.fertnstert.2016.10.02223

Flores
VA
, 
Vanhie
A
, 
Dang
T
, 
Taylor
HS
. Progesterone receptor status predicts response to progestin therapy in endometriosis. J Clin Endocrinol Metab. 2018;103:4561–4568.3035738010.1210/jc.2018-01227PMC622660224

Inoue
S
, 
Hirota
Y
, 
Ueno
T
, 
Fukui
Y
, 
Yoshida
E
, 
Hayashi
T
, et al. Uterine adenomyosis is an oligoclonal disorder associated with KRAS mutations. Nat Commun. 2019;10(1):5785. 10.1038/s41467-019-13708-y
31857578PMC692338925

Hirata
T
, 
Izumi
G
, 
Takamura
M
, 
Saito
A
, 
Nakazawa
A
, 
Harada
M
, et al. Efficacy of dienogest in the treatment of symptomatic adenomyosis: a pilot study. Gynecol Endocrinol. 2014;30:726–729.2490572510.3109/09513590.2014.92688226

Osuga
Y
, 
Fujimoto‐Okabe
H
, 
Hagino
A
. Evaluation of the efficacy and safety of dienogest in the treatment of painful symptoms in patients with adenomyosis: a randomized, double‐blind, multicenter, placebo‐controlled study. Fertil Steril. 2017;108:673–678.2891193410.1016/j.fertnstert.2017.07.02127

Neriishi
K
, 
Hirata
T
, 
Fukuda
S
, 
Izumi
G
, 
Nakazawa
A
, 
Yamamoto
N
, et al. Long‐term dienogest administration in patients with symptomatic adenomyosis. J Obstet Gynaecol Res. 2018;44:1439–1444.2984569610.1111/jog.1367428

Fawzy
M
, 
Mesbah
Y
. Comparison of dienogest versus triptorelin acetate in premenopausal women with adenomyosis: a prospective clinical trial. Arch Gynecol Obstet. 2015;292:1267–1271.2599048010.1007/s00404-015-3755-529

Hassanin
AI
, 
Youssef
AA
, 
Yousef
AM
, 
Ali
MK
. Comparison of dienogest versus combined oral contraceptive pills in the treatment of women with adenomyosis: a randomized clinical trial. Int J Gynaecol Obstet. 2021;154:263–269.3345499510.1002/ijgo.1360030

Beatty
MN
, 
Blumenthal
PD
. The levonorgestrel‐releasing intrauterine system: safety, efficacy, and patient acceptability. Ther Clin Risk Manag. 2009;5:561–574.1970727310.2147/tcrm.s5624PMC272418731

Fong
YF
, 
Singh
K
. Medical treatment of a grossly enlarged adenomyotic uterus with the levonorgestrel‐releasing intrauterine system. Contraception. 1999;60:173–175.1064016210.1016/s0010-7824(99)00075-x32

Shaaban
OM
, 
Ali
MK
, 
Sabra
AMA
, 
El Aal
DEMA
. Levonorgestrel‐releasing intrauterine system versus a low‐dose combined oral contraceptive for treatment of adenomyotic uteri: a randomized clinical trial. Contraception. 2015;92:301–317.2607167310.1016/j.contraception.2015.05.01533

Abbas
AM
, 
Samy
A
, 
Atwa
K
, 
Ghoneim
HM
, 
Lotfy
M
, 
Mohammed
HS
, et al. The role of levonorgestrel intra‐uterine system in the management of adenomyosis: a systematic review and meta‐analysis of prospective studies. Acta Obstet Gynecol Scand. 2020;99:571–581.3188929410.1111/aogs.1379834
NICE
. Heavy Menstrual Bleeding: Assessment and Management. 2018. Available from: https://www.nice.org.uk/guidance/ng88/chapter/recommendations#management‐of‐hmb. Accessed July 7 2018.35

Capmas
P
, 
Brun
JL
, 
Legendre
G
, 
Koskas
M
, 
Merviel
P
, 
Fernandez
H
. Ulipristal acetate use in adenomyosis: a randomized controlled trial. J Gynecol Obstet Hum Reprod. 2021;50:101978.3318677210.1016/j.jogoh.2020.10197836

Hong
YH
, 
Han
SJ
, 
Lee
D
, 
Kim
SK
, 
Jee
BC
. Adverse symptoms during short‐term use of ulipristal acetate in women with uterine myomas and/or adenomyosis. J Obstet Gynaecol Res. 2019;45:865–870.3067596510.1111/jog.1391737

Lupicka
M
, 
Socha
BM
, 
Szczepanska
AA
, 
Korzekwa
AJ
. Prolactin role in the bovine uterus during adenomyosis. Domest Anim Endocrinol. 2017;58:1–13.2759197910.1016/j.domaniend.2016.07.00338

Andersson
JK
, 
Khan
Z
, 
Weaver
AL
, 
Vaughan
LE
, 
Gemzell‐Danielsson
K
, 
Stewart
EA
. Vaginal bromocriptine improves pain, menstrual bleeding and quality of life in women with adenomyosis: a pilot study. Acta Obstet Gynecol Scand. 2019;98:1341–1350.3102531310.1111/aogs.1363239

Donnez
O
, 
Donnez
J
. Gonadotropin‐releasing hormone antagonist (linzagolix): a new therapy for uterine adenomyosis. Fertil Steril. 2020;114:640–645.3250731510.1016/j.fertnstert.2020.04.01740

Kavoussi
SK
, 
Esqueda
AS
, 
Jukes
LM
. Elagolix to medically treat a uterine adenomyoma: a case report. Eur J Obstet Gynecol Reprod Biol. 2020;247:266–267.3208807010.1016/j.ejogrb.2020.02.02741

Borini
A
, 
Coticchio
G
. Gonadotropin‐releasing hormone antagonist linzagolix: possible treatment for assisted reproduction patients presenting with adenomyosis and endometriosis. Fertil Steril. 2020;114:517–518.3276294910.1016/j.fertnstert.2020.06.00342

Donnez
J
, 
Dolmans
MM
. Hormone therapy for intramural myoma‐related infertility from ulipristal acetate to GnRH antagonist: a review. Reprod Biomed Online. 2020;41:431–442.3270375610.1016/j.rbmo.2020.05.01743

Singtripop
T
, 
Mori
T
, 
Park
MK
, 
Sakamoto
S
, 
Kawashima
S
. Development of uterine adenomyosis after treatment with dopamine antagonists in mice. Life Sci. 1991;49:201–206.206217510.1016/0024-3205(91)90004-u44

Badawy
AM
, 
Elnashar
AM
, 
Mosbah
AA
. Aromatase inhibitors or gonadotropin‐releasing hormone agonists for the management of uterine adenomyosis: a randomized controlled trial. Acta Obstet Gynecol Scand. 2012;91:489–495.2222925610.1111/j.1600-0412.2012.01350.x45

Liu
X
, 
Shen
M
, 
Qi
Q
, 
Zhang
H
, 
Guo
SW
. Corroborating evidence for platelet‐induced epithelial‐mesenchymal transition and fibroblast‐to‐myofibroblast transdifferentiation in the development of adenomyosis. Hum Reprod. 2016;31:734–749.2690884510.1093/humrep/dew01846

Huang
JH
, 
Duan
H
, 
Wang
S
, 
Wang
YY
, 
Lv
CX
. Upregulated microRNA let‐7a accelerates apoptosis and inhibits proliferation in uterine junctional zone smooth muscle cells in adenomyosis under conditions of a normal activated Hippo‐YAP1 axis. Reprod Biol Endocrinol.
2021;19(1):81.3408277410.1186/s12958-021-00753-wPMC8173847

### 2.3 Surgical management

Adenomyosis has variable clinical presentations of dysmenorrhea, HMB, chronic pelvic pain, and subfertility depending, perhaps in no small part, on the location, extent, and composition of the lesion. On top of these wide variations, there is an extensive variation in patients’ age, socioeconomic status, reproductive history, presence or absence of comorbidity, and their desire and wishes. As such, any treatment should be individualized, and tailored to match the patient's wishes. There is no treatment for adenomyosis for women who desire to retain their uteri or wish to preserve fertility. Medical treatment is usually the first choice, whereas surgery can be a viable option for refractory adenomyosis or those who are unsuitable for long‐term medical treatment. However, combined treatment can be considered: Laparoscopy, GnRHa treatment, assisted reproductive techniques, and levonorgestrel‐releasing intrauterine system.^1^, ^2^, ^3^, ^4^, ^5^ To spare the uterus, there are many procedures to relieve symptoms including nonexcisional and excisional techniques (the partial and complete excision of the lesion). The ultimate treatment, if all fails, is hysterectomy, which is the most effective way of achieving symptom control and provides high satisfaction rates for patients who have no fertility need or no desire to keep their uterus.

#### 2.3.1 Nonexcisional techniques

There are several nonexcisional techniques for adenomyosis treatment, including endometrial/endomyometrial ablation, electrocoagulation, uterine artery embolization (UAE), and ablation by radiofrequency, microwave, and high‐intensity focused ultrasound (HIFU). They have been applied to control the symptoms, prevent early recurrences, and offer a desirable uterine environment for implantation and pregnancy.^6^ After endomyometrial ablation, the percentage of women who had dysmenorrhea and HMB decreased from 70% to 33% and 86% to 14%, respectively.^7^, ^8^ UAE has also been described as a promising treatment of symptoms resulting from adenomyosis. However, it can affect both hormonal production and ovarian reserve, leading to premature ovarian failure and iatrogenic HMB and infertility. Endometrial receptivity is also diminished after this procedure. Therefore, it should be contraindicated in women planning a pregnancy but may be useful in the postreproductive age.^9^ Electrocoagulation has also been applied to focal or diffuse diseases. However, the main disadvantage of electrocoagulation is that it may be less accurate than surgical excision, as well as poorly controlled during the procedure. There are two types of HIFU, ultrasound‐guided HIFU (USgHIFU)^10^ and magnetic resonance‐guided focused ultrasound surgery (MRgFUS).^11^ Both methods employ the thermal effect of the focused ultrasound beam, which causes coagulative necrosis within the targeted adenomyotic lesion. The lesion should be visible in ultrasound or under MRI so that the beam could be precisely directed. The advantages of USgHIFU over MRgFUS are a shorter treatment time, relatively lower cost, and a higher nonperfused volume ratio. HIFU may face challenges for the diffuse form of adenomyosis. In addition, since external/extrinsic adenomyosis is closely associated with deep endometriosis^12^ and since HIFU can only ablate adenomyotic, but not endometriotic, lesions, HIFU treatment is likely unable to completely remove all sources for symptoms—at least as of now. Moreover, the recurrence risk appears to be high. Of the most concerning is the fact that, most, if not all, published studies on HIFU, radiofrequency and microwave ablations are retrospective, but surprisingly not a single RCT yet, caution should be exercised. Last, but not least, there has been no study that compares, head‐to‐head, efficacy between HIFU, or any other ablation method, and other procedural treatment, such as hysterectomy and adenomyomectomy.^13^

#### 2.3.2 Excisional techniques

Uterine‐sparing excisional techniques for adenomyosis can be divided into complete excision of adenomyosis (adenomyomectomy) for focal adenomyosis (adenomyoma) and partial excision of adenomyosis (cytoreductive surgery) for extensive adenomyosis (diffuse adenomyosis) in which the removal of visible lesions is only partial. Of course, in the hands of experienced and skilled surgeons, adenomyomectomy can be performed for both focal and diffuse adenomyosis. The techniques are similar to that of myomectomy and can be performed by laparoscopy or laparotomy. The justification for performing extensive surgery beyond that is like myomectomy remains unclear.^14^ Caution should be taken to minimize the risk of unintended removal of normal myometrial tissues. For complete excision of adenomyotic lesions, the lesion is separated from the normal myometrium and excised but the plane between adenomyoma and normal myometrium is not well demarcated. Partial excision of adenomyosis for diffuse adenomyosis requires massive removal of adenomyotic foci including a large amount of healthy myometrium. The removal of visible lesions is only partial because further tissue excision could lead to a “functional hysterectomy”.^8^

Most excisional techniques can be performed by laparoscopy or laparotomy. To reduce intraoperative blood loss, injection of vasopressin solution into the myometrium or concomitant uterine artery occlusion has been advocated. Incisions on the uterine wall could be transverse, vertical, diagonal, H incisions (1 vertical and 2 horizontal incisions), and variation of excision could be classical excision of adenomyotic tissue after longitudinal incision of the uterus, wedge resection,^3^, ^15^ a variation of the flap method,^15^, ^16^, ^17^, ^18^ and *U*‐shaped resection of the adenomyotic tissue,^18^ depending on location and individual preference of the surgeon. The fallopian tubes should be left patent to allow spontaneous pregnancy. To prevent uterine rupture, the removal of significant amounts of the myometrium with the adenomyotic lesion should be avoided.^19^, ^20^ The uterine defect should be reconstructed thoroughly with meticulous suturing without leaving any dead space in the fashions of U‐shaped suturing, multilayer suturing, or overlapping flap technique. The recommended suture material is barbed suture.^21^ The seromuscular layer is closed with a figure of 8 sutures, the overlapping flaps technique, double‐flap, or the triple‐flap method.^16^, ^17^, ^19^, ^22^, ^23^ Finally, the uterine incision is covered with an adhesion barrier to reduce adhesion formation.

#### 2.3.3 Uterus‐sparing surgical outcomes

Endomyometrial resection is effective and indicated in patients with lesions confined to the endomyometrial junction and alleviation of HMB6. However, in patients who desire pregnancy, endomyometrial resection is contraindicated.^24^ A systematic review and meta‐analysis evaluated the outcome of conservative surgery for adenomyosis.^8^ The clinical outcome at the follow‐up of 12 months was selected for presenting the parameters under investigation. Five studies (*n* = 612 patients) reported complete excision of adenomyosis (adenomyomectomy).^16^, ^17^, ^25^, ^26^, ^27^ The postoperative measurement of pain and menorrhagia improved by 70% to 90% and 70% to 92%, respectively, and the reduction of uterine volume was reduced by 65%. Seven studies (*n* = 559 patients) reported uterus‐sparing treatment of adenomyosis with partial excision of adenomyosis.^18^, ^28^, ^29^, ^30^, ^31^, ^32^, ^33^ A hysteroscopic endomyometrial approach under ultrasound guidance was included in this group.^30^ After partial excision of adenomyosis, the postoperative measurement of pain and menorrhagia improved by 41% to 90% and 48% to 89%, respectively, and the reduction of uterine volume was reduced by 25% to 87%. The studies with a mixed volume of patients with complete and partial excision of adenomyosis reported improvement in pain, menorrhagia, and reduction of uterine volume by a factor of 4.0, 6.3, and 5.1, respectively. Another systemic review found that intraoperative blood loss varied widely, from 30 to 80 mL in laparoscopic adenomyomectomy with or without uterine artery occlusion to 370 to 400 mL in the double‐flap and triple‐flap methods.^14^

Regarding fertility outcomes, destruction of the endometrium together with the junctional zone can cause serious complications in patients who managed to conceive, such as miscarriage, preterm labor, and placentation complications.^8^ An unexpectedly high rate of pregnancy complications after endometrial ablation is reported in a systemic review.^34^ After high‐intensity focused ultrasound, published data indicates that patients can attempt to conceive much earlier than after surgical treatment, but the exact time of delay in conception is unknown and the rate of uterine ruptures during pregnancy or delivery is lower than after classical surgical methods. Although the miscarriage rate appears to be quite high after the high‐intensity focused ultrasound method, other severe complications like uterine rupture did not occur.^10^ However, the myometrial tissue is affected, which may reduce the strength of the uterine wall and induce a risk of rupture in pregnancy. No larger studies on pregnancy outcomes and only cases of pregnancy are reported after these procedures for adenomyosis.^35^, ^36^ At present, these techniques have, therefore, not been recommended for women with adenomyosis and a wish to conceive.

A recent meta‐analysis concluded that conservative surgery in adenomyosis could improve fertility in some patients, but the rate of successful pregnancies varied among surgical centers^14^), suggesting that the success rate may hinge tightly on the skill levels of the surgeon. Eleven studies evaluated fertility outcomes with pregnancy rates varying between studies (25%–100%) and live birth rates of 32%–100%. Complete excision resulted in a higher pregnancy rate of up to 100% versus 50% in incomplete excision. The highest pregnancy rates were found in complete excision of cystic adenomyomas. There were 2 cases of uterine rupture at 32 and 37 weeks of gestation in women who had undergone a wedge resection of adenomyosis uterus, and the importance of meticulous uterine closure is emphasized.^19^ In another study,^20^ 2 of 23 pregnancies after cytoreductive surgery had ruptured in the second trimester. Only 2 in 5 women with myometrial thickness <7 mm had normal pregnancies. The authors concluded that the optimal wall thickness for conception and prevention of rupture after cytoreductive surgery may range from 9 to 15 mm.^20^

Another meta‐analysis concluded that the conception rates after uterus‐sparing surgery for adenomyosis appear to be satisfactory. Conception, full‐term, and total delivery rates after complete excision of adenomyosis were 26.9%, 76.7%, and 85.1%, respectively, in a study of 71 women, three‐fourths of them conceived after surgery with or without adjuvant medical treatment,^37^ whereas, conception, full‐term, and total delivery rates after partial excision were 50.0%, 66.7%, and 73.3%, respectively. Moreover, early pregnancy wastage does not seem to be increased, pregnancies seem to continue without significant complications, and the viable term delivery rates seem to be satisfactory.^9^ Morbid variations of placentation rates (placenta previa, placental percreta) do not seem to be increased. Moreover, a nonsystematic review described 23 cases of uterine rupture out of 2365 women who underwent adenomyomectomy (1.0%).^38^ The author concluded that uterine rupture after uterus‐sparing surgical treatment of adenomyosis seems to be related to the removal of adenomyotic tissue technique, the degree of remnants of adenomyosis left postoperatively, the uterine wall thickness, postoperative complications (infection or hematoma), and the interval between the procedure and conception. Cesarean section is usually a preferred delivery route after adenomyosis excision treatment.

#### 2.3.4 Uterus‐sparing surgical complication

Excision of extensive adenomyosis is difficult and associated with a high complication and high recurrence rate if performed by inexperienced surgeons. The complication rate is usually associated with many factors such as surgical skill and experience, surgical approaches, type and severity of adenomyosis, etc. The common complications were reported such as intraoperative blood transfusion, postoperative fever, hematomas, and intrauterine adhesion after wedge resection of adenomyosis, cervical tears during hysteroscopy.^14^ A meta‐analysis study^8^ found that common complications in this surgical technique were blood loss (36−372 mL), uterine hematomas (6/1843, or 0.3%), and febrile morbidity (10/1843, or 0.5%) and hysterectomy (22/1843, or 1.2%). Three in 1843 cases (0.2%) had serious surgical complications: small bowel perforation, epigastric artery bleeding at the trocar site, and ileus. Hysterectomy has finally performed in 1.2% (*n* = 22/1843), 5/612 (0.8%) cases who had complete excision, 6/559 (1.1%) cases who had partial excision, 3/373 (0.8%) cases who had nonspecific excision, and 8/43 (18/6%) cases who had endometrial ablation.^8^ Regarding the differences in excision surgical technique, flap approaches are not associated with extra morbidity, and no reports are indicating that hematomas, postoperative dehiscence of the uterine scar, or adhesions are increased, either after laparotomy or laparoscopy. However, the authors suggest that these findings should be interpreted under the light of surgical experience because these results are reported from centers of surgical excellence, where extensive surgical experience in all surgical techniques increases the possibility of a good postoperative outcome. Moreover, the variations of surgical approaches should be considered, as how similar are the “triple‐flap” and “double‐flap” methods in terms of tissue excision and, more importantly, tissue restoration. Studies specifically designed to answer these questions are still not available. There has been no recommendation for a compulsory waiting time to conceive after surgery for adenomyosis. According to standard recommendations after a myomectomy, the authors suggested a waiting time of at least 3 months between surgery and the attempt to conceive.^8^

#### 2.3.5 Recurrence

Depending on the duration of follow‐up, the published recurrence rates differ from no recurrence at all to almost half of the patients. The best symptom improvement is in the first year after surgery. Adenomyosis recurrence by ultrasound was reported to be 15% in 27 months after surgery.^39^ Recurrences of adenomyosis, in the span of 12–123 months after surgery, were reported in 3.3% [*n* = 60/1843, 35/612 (6.0%) cases who had complete excision, 14/559 (2.5%) cases who had partial excision, and 11/ 43 (25.5%) cases who had endometrial ablation].^8^ Comparable to another systemic review, the lowest rate of recurrence is after complete excision and the highest after nonexcisional techniques.^14^

#### 2.3.6 Hysterectomy

Hysterectomy is still the only definitive treatment for patients with adenomyosis. However, it causes many adverse effects and is not suitable for patients who wish to remain fertile. Three studies (*n* = 256 patients) were included in a systemic review that reported treatment of adenomyosis with hysterectomy.^13^, ^40^, ^41^ After the hysterectomy, the postoperative measurement of pain improved by 84%. It is associated with an improvement in pain by a factor of 2.2. A prospective observational study showed that laparoscopic supracervical hysterectomy in women with perioperative detection of endometriosis or histologic confirmation of adenomyosis is associated with high patient satisfaction and reduces cyclic pelvic pain to a minimum by 12 months after the procedure. A retrospective cohort study found that following surgery, women with adenomyosis were less likely than those without adenomyosis to report persistent pain (adjusted OR = 0.43; 95% CI = 0.20–0.93; *p* = 0.03).^40^

#### 2.3.7 Conclusion

In many cases, women with adenomyosis are treated with medication. With an acceptable complication rate, uterine‐sparing surgery is offered to women with refractory adenomyosis or unsuitable for long‐term medical treatment and can improve pelvic pain, HMB, and possibly fertility. Most nonexcisional techniques are still restricted to women who have fertility needs. Excisional techniques are similar to that of myomectomy and can be performed by laparoscopy or laparotomy. The justification for performing extensive surgery, like myomectomy, remains unclear. The best method of uterine‐sparing surgery is yet to be demonstrated because most systemic reviews did not include randomized controlled trials, and with the published studies there is extensive heterogeneity regarding surgical skills, surgical procedures, and the type of instruments used to quantify the pain, and the bleeding. Moreover, there are differences between the studies in terms of follow‐up and rates of lost patients during re‐examinations.

There is no consensus on optimal treatment for patients with adenomyosis who want to retain their uterus or wish to preserve fertility.^1^ Sometimes, combined treatment can be proposed: Laparoscopy, GnRHa treatment, and in vitro fertilization. When comparing pharmacological and surgical treatment, the latter appears to be more effective, but some details are unclear, that is, how long pregnancy should be delayed after treatment and whether hormone treatment after surgery improves fertility outcomes. Apparently, head‐to‐head comparison trials can be very challenging to conduct, especially in a random assignment and double‐blinded fashion. Despite many studies on the pathogenesis of fertility failure in adenomyosis, their results are not correlated with treatment. Thus, it is of great importance to explore new, more effective, safe, and less invasive managing strategies in women with infertility due to adenomyosis.

REFERENCES1

Vannuccini
S
, 
Petraglia
F
. Recent advances in understanding and managing adenomyosis. F1000Research. 2019;8. F1000 Faculty Rev‐283. 10.12688/f1000research.17242.1
PMC6419978309186292

Soritsa
D
, 
Saare
M
, 
Laisk‐Podar
T
, 
Peters
M
, 
Soritsa
A
, 
Matt
K
, et al. Pregnancy rate in endometriosis patients according to the severity of the disease after using a combined approach of laparoscopy, GnRH agonist treatment and in vitro fertilization. Gynecol Obstet Invest. 2015;79(1):34–39.2527780210.1159/0003653293

Lin
CJ
, 
Hsu
TF
, 
Chang
YH
, 
Huang
BS
, 
Jiang
LY
, 
Wang
PH
, et al. Postoperative maintenance levonorgestrel‐releasing intrauterine system for symptomatic uterine adenomyoma. Taiwan J Obstet Gynecol. 2018;57(1):47–51.2945890210.1016/j.tjog.2017.12.0364

Koo
YJIK
, 
Kwon
YS
. Conservative surgical treatment combined with GnRH agonist in symptomatic uterine adenomyosis. Pak J Med Sci. 2011;27:365–370.5

Dueholm
M
. Uterine adenomyosis and infertility, review of reproductive outcome after in vitro fertilization and surgery. Acta Obstet Gynecol Scand. 2017;96:715–726.2855612410.1111/aogs.131586

Busca
A
, 
Parra‐Herran
C
. The role of pathologic evaluation of endometrial ablation resections in predicting ablation failure and adenomyosis in hysterectomy. Pathol Res Pract. 2016;212:778–782.2746182310.1016/j.prp.2016.06.0077

Philip
CA
, 
Le Mitouard
M
, 
Maillet
L
, 
de Saint‐Hilaire
P
, 
Huissoud
C
, 
Cortet
M
, et al. Evaluation of NovaSure global endometrial ablation in symptomatic adenomyosis: a longitudinal study with a 36‐month follow‐up. Eur J Obstet Gynecol Reprod Biol. 2018;227:46–51.2988631710.1016/j.ejogrb.2018.04.0018

Mikos
T
, 
Lioupis
M
, 
Anthoulakis
C
, 
Grimbizis
FG
. The outcome of fertility‐sparing and nonfertility‐sparing surgery for the treatment of adenomyosis. A systematic review and meta‐analysis. J Minim Invasive Gynecol. 2020;27(2):309–331.e3. 10.1016/j.jmig.2019.08.004
313984159

De Bruijn
AM
, 
Smink
M
, 
Lohle
PN
, 
Huirne
JA
, 
Twisk
JW
, 
Wong
C
, et al. Uterine artery embolization for the treatment of adenomyosis: a systematic review and meta‐analysis. J Vasc Interv Radiol. 2017;28:1629–1642.2903294610.1016/j.jvir.2017.07.03410

Zhang
L
, 
Rao
F
, 
Setzen
R
. High intensity focused ultrasound for the treatment of adenomyosis: selection criteria, efficacy, safety and fertility. Acta Obstet Gynecol Scand. 2017;96:707–714.2843700310.1111/aogs.1315911

Zowall
H
, 
Cairns
JA
, 
Brewer
C
, 
Lamping
DL
, 
Gedroyc
WM
, 
Regan
L
. Cost‐effectiveness of magnetic resonance‐guided focused ultrasound surgery for treatment of uterine fibroids. BJOG. 2008;115:653–662.1833394810.1111/j.1471-0528.2007.01657.xPMC234416212

Bourdon
M
, 
Oliveira
J
, 
Marcellin
L
, 
Santulli
P
, 
Bordonne
C
, 
Maitrot Mantelet
L
, et al. Adenomyosis of the inner and outer myometrium are associated with different clinical profiles. Hum Reprod.
2021;36(2):349–357.3349105710.1093/humrep/deaa30713

Liu
XF
, 
Huang
LH
, 
Zhang
C
, 
Huang
GH
, 
Yan
LM
, 
He
J
. A comparison of the cost‐utility of ultrasound‐guided high‐intensity focused ultrasound and hysterectomy for adenomyosis: a retrospective study. BJOG. 2017;124(Suppl 3):40–45.2885686610.1111/1471-0528.1474614

Younes
G
, 
Tulandi
T
. Conservative surgery for adenomyosis and results: a systematic review. J Minim Invasive Gynecol. 2018;25(2):265–276. 10.1016/j.jmig.2017.07.014
2873941415

Kitade
M
, 
Jinushi
M
, 
Ikuma
S
, 
Murakami
K
, 
Ozaki
R
, 
Masuda
A
, et al. Surgical treatments for adenomyosis. Uterine fibroids and adenomyosis. Comprehensive gynecology and obstetrics. Singapore: Springer; 2018. p. 151–162.16

Chong
GO
, 
Lee
YH
, 
Hong
DG
, 
Cho
YL
, 
Lee
YS
. Long‐term efficacy of laparoscopic or robotic adenomyomectomy with or without medical treatment for severely symptomatic adenomyosis. Gynecol Obstet Invest. 2016;81:346–352.2689448810.1159/00044178317

Osada
H
, 
Silber
S
, 
Kakinuma
T
, 
Nagaishi
M
, 
Kato
K
, 
Kato
O
. Surgical procedure to conserve the uterus for future pregnancy in patients suffering from massive adenomyosis. Reprod Biomed Online. 2011;22:94–99.2111875110.1016/j.rbmo.2010.09.01418

Huang
X
, 
Huang
Q
, 
Chen
S
, 
Zhang
J
, 
Lin
K
, 
Zhang
X
. Efficacy of laparoscopic adenomyomectomy using double‐flap method for diffuse uterine adenomyosis. BMC Womens Health. 2015;15:24. 10.1186/s12905-015-0182-5
25783654PMC435949819

Saremi
A
, 
Bahrami
H
, 
Salehian
P
, 
Hakak
N
, 
Pooladi
A
. Treatment of adenomyomectomy in women with severe uterine adenomyosis using a novel technique. Reprod Biomed Online. 2014;28:753–760.2476855810.1016/j.rbmo.2014.02.00820

Otsubo
Y
, 
Nishida
M
, 
Arai
Y
, 
Ichikawa
R
, 
Taneichi
A
, 
Sakanaka
M
. Association of uterine wall thickness with pregnancy outcome following uterine‐sparing surgery for diffuse uterine adenomyosis. Aust N Z J Obstet Gynaecol. 2016;56(1):88–91.2651593610.1111/ajo.1241921

Tulandi
T
, 
Einarsson
JI
. The use of barbed suture for laparoscopic hysterectomy and myomectomy: a systematic review and meta‐analysis. J Minim Invasive Gynecol. 2014;21:210–216.2412625710.1016/j.jmig.2013.09.01422

Sun
AJ
, 
Luo
M
, 
Wang
W
, 
Chen
R
, 
Lang
JH
. Characteristics and efficacy of modified adenomyomectomy in the treatment of uterine adenomyoma. Chin Med J. 2011;124:1322–1326.2174074123

Takeuchi
H
, 
Kitade
M
, 
Kikuchi
I
, 
Shimanuki
H
, 
Kumakiri
J
, 
Kitano
T
, et al. Laparoscopic adenomyomectomy and hysteroplasty: a novel method. J Minim Invasive Gynecol.
2006;13(2):150–154.1652771910.1016/j.jmig.2005.12.00424

Wood
C
. Adenomyosis: difficult to diagnose, and difficult to treat. Diagn Ther Endosc. 2001;7:89–95.1849355210.1155/DTE.7.89PMC236283325

Kwack
JY
, 
Im
KS
, 
Kwon
YS
. Conservative surgery of uterine adenomyosis via laparoscopic versus laparotomic approach in a single institution. J Obstet Gynaecol Res. 2018;44:1268–1273.2984568710.1111/jog.1365826

Dai
Z
, 
Feng
X
, 
Gao
L
, 
Huang
M
. Local excision of uterine adenomyomas: a report of 86 cases with follow‐up analyses. Eur J Obstet Gynecol Reprod Biol. 2012;161:84–87.2215390410.1016/j.ejogrb.2011.11.02827

Wang
PH
, 
Liu
WM
, 
Fuh
JL
, 
Cheng
MH
, 
Chao
HT
. Comparison of surgery alone and combined surgical‐medical treatment in the management of symptomatic uterine adenomyoma. Fertil Steril. 2009;92:876–885.1877456610.1016/j.fertnstert.2008.07.174428

Jun‐Min
X
, 
Kun‐Peng
Z
, 
Yin‐Kai
Z
, 
Ya‐Qin
Z
, 
Xiao‐Fan
F
, 
Xiao‐Yu
Z
, et al. A new surgical method of U‐shaped myometrial excavation and modified suture approach with uterus preservation for diffuse adenomyosis. Biomed Res Int. 2018;2018:1657237. 10.1155/2018/1657237
30112362PMC607767329

Yu
W
, 
Liu
G
, 
Liu
C
, 
Zhang
Z
. Recurrence‐associated factors of laparoscopic adenomyomectomy for severely symptomatic adenomyoma. Oncol Lett. 2018;16:3430–3438.3012794510.3892/ol.2018.9082PMC609615230

Xia
W
, 
Zhang
D
, 
Zhu
Q
, 
Zhang
H
, 
Yang
S
, 
Ma
J
, et al. Hysteroscopic excision of symptomatic myometrial adenomyosis: feasibility and effectiveness. BJOG. 2017;124(10):1615–1620.2854426010.1111/1471-0528.1467331

Yang
B
, 
Wang
L
, 
Wan
X
, 
Li
Y
, 
Yu
X
, 
Qin
Y
, et al. Elevated plasma levels of lysophosphatidic acid and aberrant expression of lysophosphatidic acid receptors in adenomyosis. BMC Womens Health. 2017;17(1):118.2917892210.1186/s12905-017-0474-zPMC570223432

Wang
PH
, 
Fuh
JL
, 
Chao
HT
, 
Liu
WM
, 
Cheng
MH
, 
Chao
KC
. Is the surgical approach beneficial to subfertile women with symptomatic extensive adenomyosis?
J Obstet Gynaecol Res. 2009;35:495–502.1952738910.1111/j.1447-0756.2008.00951.x33

Kang
L
, 
Gong
J
, 
Cheng
Z
, 
Dai
H
, 
Liping
H
. Clinical application and midterm results of laparoscopic partial resection of symptomatic adenomyosis combined with uterine artery occlusion. J Minim Invasive Gynecol. 2009;16:169–173.1924970410.1016/j.jmig.2008.12.00334

Kohn
JR
, 
Shamshirsaz
AA
, 
Popek
E
, 
Guan
X
, 
Belfort
MA
, 
Fox
KA
. Pregnancy after endometrial ablation: a systematic review. BJOG. 2018;125:43–53.2895218510.1111/1471-0528.1485435

Kim
MD
, 
Kim
NK
, 
Kim
HJ
, 
Lee
MH
. Pregnancy following uterine artery embolization with polyvinyl alcohol particles for patients with uterine fibroid or adenomyosis. Cardiovasc Intervent Radiol. 2005;28:611–615.1613238510.1007/s00270-004-8236-336

Lee
JS
, 
Hong
GY
, 
Park
BJ
, 
Kim
TE
. Ultrasound‐guided high‐intensity focused ultrasound treatment for uterine fibroid & adenomyosis: a single center experience from the Republic of Korea. Ultrason Sonochem. 2015;27:682–687.2607236710.1016/j.ultsonch.2015.05.03337

Maia
H
Jr
, 
Maltez
A
, 
Coelho
G
, 
Athayde
C
, 
Coutinho
EM
. Insertion of mirena after endometrial resection in patients with adenomyosis. J Am Assoc Gynecol Laparosc. 2003;10:512–516.1473864010.1016/s1074-3804(05)60158-238

Osada
H
. Uterine adenomyosis and adenomyoma: the surgical approach. Fertil Steril. 2018;109:406–417.2956685310.1016/j.fertnstert.2018.01.03239

Hyams
LL
. Adenomyosis; its conservative surgical treatment (hysteroplasty) in young women. N Y State J Med. 1952;52:2778–2784.1300288240

Ajao
MO
, 
Oliveira Brito
LG
, 
Wang
KC
, 
Cox
MKB
, 
Meurs
E
, 
Goggins
ER
, et al. Persistence of symptoms after total vs supracervical hysterectomy in women with histopathological diagnosis of adenomyosis. J Minim Invasive Gynecol. 2019;26(5):891–896.3020516410.1016/j.jmig.2018.09.00241

Berner
E
, 
Qvigstad
E
, 
Myrvold
AK
, 
Lieng
M
. Pelvic pain and patient satisfaction after laparoscopic supracervical hysterectomy: prospective trial. J Minim Invasive Gynecol. 2014;21:406–411.2417745210.1016/j.jmig.2013.10.011

## 3 CLINICAL QUESTIONS (CQS)

### 3.1 Reference selection and CQ assessment

The committee members and authors first selected the keywords and the main papers associated with the CQs. To assess the evidence at the present stage from an impartial perspective, we requested the Japan Medical Library Association to conduct a comprehensive literature search by creating different queries for each CQ. PubMed was searched for studies published from 2017 to 2021. The selection criteria were as follows: Practice guidelines, meta‐analyses, and systematic reviews were given the highest priority. Subsequently, RCT, prospective cohort studies, retrospective studies, clinical trials, and other epidemiological studies were selected.

To rank the level of evidence and recommendations based on a symptom prevalence study, we used the Oxford Centre for Evidence‐Based Medicine (CEBM) criteria for Levels of Evidence and Grade of Recommendation, as explained below.

Levels of Evidence:

1a: Systematic review (with homogeneity) of prospective cohort studies (RCT)

1b: Prospective cohort study with good follow‐up

Individual RCT (with narrow confidential interval)

1c: All or noncase series

2a: Systematic review (with homogeneity) of 2b and better studies

2b: Retrospective cohort study or poor follow‐up

2c: Ecological studies

3a: Systematic review (with homogeneity) of 3b and better studies

3b: Nonconsecutive cohort study, or very limited population

4: Case series or superseded reference standards

5: Expert opinion without an explicit critical appraisal, or based on physiology, bench research, or “first principles”

Grade of Recommendation:

A: Consistent level 1 studies

B: Consistent level 2 or 3 studies or extrapolations from level 1 studies

C: Level 4 studies or extrapolations from level 2 or 3 studies

D: Level 5 evidence or troubling inconsistent or inconclusive studies at any level

The committee members and the authors reached a consensus through discussion on the assessment of each CQ.

### 3.2 CQ1: Is imaging useful for diagnosis of adenomyosis?

Transvaginal ultrasound (TVUS) and Magnetic Resonance Imaging (MRI) are good noninvasive methods of diagnosing adenomyosis.

**Table:** 

Level of evidence	1a
Grade of recommendation	A

TVUS should be considered the first‐line diagnostic method while MRI is recommended as a second‐line method when TVUS is inconclusive.

**Table:** 

Level of evidence	1a
Grade of recommendation	A

Most diagnostic features of adenomyosis could be demonstrated using two‐dimensional (2D) TVUS and the addition of three‐dimensional (3D) TVUS will not increase the diagnostic accuracy significantly.

**Table:** 

Level of evidence	1a
Grade of recommendation	A

Transabdominal ultrasound is of limited value but may be of use when TVUS is not possible or with grossly enlarged uteri. The method has a low specificity (30%) compared to TVUS (up to 100%).

**Table:** 

Level of evidence	4
Grade of recommendation	C

Further research is required regarding the place of Elastography in the diagnosis of adenomyosis.

**Table:** 

Level of evidence	3b
Grade of recommendation	C

Meta‐analyses – 3

Reviews – 3

Expert opinions – 2

#### 3.2.1 Commentary

Imaging is the cornerstone in the modern‐day diagnosis of adenomyosis. It has revolutionized the process from one that is based on histology to one that is noninvasive. Accurate preoperative diagnosis, mapping of lesions, and assessment of severity are now possible using imaging. Most women suffering adenomyosis today will be treated conservatively, without the need for histological proof.

TVUS and MRI are the main modalities of imaging used. They are considered comparable noninvasive diagnostic methods in adenomyosis.^1^, ^2^, ^3^

#### 3.2.2 TVUS

TVUS is recommended as the first line of imaging.^1^, ^2^, ^3^, ^4^ It is relatively cheap and widely available in an outpatient setting. It allows the dynamic exploration of the pelvic anatomy, examining probe tenderness and organ mobility.

The diagnostic accuracy of ultrasound is high with a sensitivity of 82.5% (95% CI = 77.5–87.9), a specificity of 84.6% (95% CI = 79.8–89.8), a positive likelihood ratio of 4.7 (3.1–7.0) and a negative likelihood ratio of 0.26 (0.18–0.39).^5^ The addition of 3D mode may not add significantly to the diagnostic accuracy.^6^

However, the area still evolving, with the lack of a common nomenclature and definitions. The MUSA terminology published by the Morphology Uterus Sonographic Assessment (MUSA) Group aims to provide a standardized terminology of normal and pathological myometrium.^7^

A summary of appearances as described by the MUSA consensus group is given in Tables 1 and 2. The first two are referred to as “indirect” signs and the rest as “direct” signs^7^

**Table 1:** Features considered important in diagnosing adenomyosis.

Sign	Mode	Definition
Asymmetry of the anterior and posterior walls	2D	A ratio above 1
Enlarged globular‐shaped uterus with globular contour	2D	Visual assessment; Exclude uterine contractions
Intramyometrial cysts	2D	Round‐shaped lesions within the myometrium, with anechoic, low‐level echogenicity, ground‐glass appearance, or mixed echogenicity of intracystic content. Typically, there is a hyperechogenic rim surrounding the cyst
Hyperechogenic islands	2D	The presence of regular, irregular ill‐defined hyperechogenic areas within the myometrium
Fan‐shaped shadowing	2D	Alternating hypoechogenic and hyperechogenic linear stripes crossing the uterine wall.
Hyperechogenic subendometrial lines or buds	2D	Structures perpendicular to the endometrial cavity, but in continuum with the endometrium
Interrupted junctional zone	3D	Best seen by rendering the coronal plane. May be irregular, interrupted, not visible, or measurable
Translesional vascularity’	Doppler (Color/power)	Helps in differentiating between a myoma and adenomyosis, by the presence of circumferential flow in a myoma, against “translesional” flow

*Note*: More recently a new reporting system was described to include further characteristics of the disease.^8^ This is described in Table 2.

**Table 2:** Classification and reporting for sonographic features of adenomyosis.

Location	Anterior Posterior Right lateral Left lateral Fundal	Described according to location
Distribution	Focal	25% or more of the lesion is surrounded by normal endometrium. When focal adenomyosis is formed by invasion from outside to inside, it is referred to as Focal Adenomyosis of the Outer Myometrium (FOAM)
	Adenomyoma	The lesion is demarcated distinctly and is totally surrounded by hypertrophic myometrium
	Diffuse	
	Mixed	Both focal and diffuse disease is present
Presence of cysts	Cystic/noncystic	Presence or absence of intramyometrial cysts measuring 2 mm or more
Layer of uterine involvement	Type 1	Involvement of the junctional zone
	Type 2	Involvement of the middle myometrium
	Type 3	Involvement of the outer myometrium. Demarcation between the middle and outer myometrium is determined by using color Doppler to delineate the vascular arcade
	Multiple layers	Described as Type 1‐2, 1‐3, etc.
Extent	Mild	<25% affected
	Moderate	25%–50% affected
	Severe	>50% affected
Lesion size	Lesion/s is/are measured in their longest diameter/s	

#### 3.2.3 MRI

The sensitivity and specificity of MRI in diagnosing adenomyosis are 88%–93% and 61%–97%, respectively.^3^ The MRI diagnosis of adenomyosis hinges mainly on the characteristics of the junctional zone (JZ), but it may include direct and indirect features described for ultrasound as well.^9^

Thickening of the JZ at least 8–12 mm, the ratio of junctional zone maximum/total myometrial thickness measured at the same point over 40%, and the difference between the maximum and the minimum thickness of the JZ (JZ_max_–JZ_min_) more than 5 mm are three of these features.^10^ A thickness exceeding 12 mm seems to be highly predictive of adenomyosis while a JZ less than 8 mm generally allows the presence of adenomyosis to be excluded.^11^

Similar to TVUS, classifications have been proposed for adenomyosis based on adenomyosis.

#### 3.2.4 Elastography

There is a suggestion that elastography may be superior to TVUS for the differentiation of fibroids and adenomyosis.^11^ However, more research is needed regarding its place in the diagnosis of adenomyosis. In particular, its sensitivity and specificity, as well as the intra‐ and interoperator variations need to be quantitated, especially given the fact that there are at least two categories of ultrasound elastography, one is based on shear‐wave and the other, strain elastography.

REFERENCES1

Tellum
T
, 
Nygaard
S
, 
Lieng
M
. Noninvasive diagnosis of adenomyosis: a structured review and meta‐analysis of diagnostic accuracy in imaging. J Minim Invasive Gynecol.
2020;27(2):408–418.e3. 10.1016/j.jmig.2019.11.001
317121622

Liu
L
, 
Li
W
, 
Leonardi
M
, 
Condous
G
, 
Da Silva
CF
, 
Mol
BW
, et al. Diagnostic accuracy of transvaginal ultrasound and magnetic resonance imaging for adenomyosis: systematic review and meta‐analysis and review of sonographic diagnostic criteria. J Ultrasound Med. 2021;40(11):2289–2306. 10.1002/jum.15635
335027673

Chapron
C
, 
Vannuccini
S
, 
Santulli
P
, 
Abro
MP
, 
Carmona
F
, 
Fraser
IS
, et al. Diagnosing adenomyosis: an integrated clinical and imaging approach. Hum Reprod Update. 2020;3:392–411.10.1093/humupd/dmz049320974564

Bazot
M
, 
Darai
E
. Role of transvaginal sonography and magnetic resonance imaging in the diagnosis of uterine adenomyosis. Fertil Steril. 2018;109:389–397.2956685110.1016/j.fertnstert.2018.01.0245

Meredith
S
, 
Sanchez‐Ramos
L
, 
Kaunitz
A
. Diagnostic accuracy of transvaginal sonography. Am J Obstet Gynecol. 2009;201:107.e1–107.e6.10.1016/j.ajog.2009.03.021193980896

Andres
MP
, 
Borrelli
GM
, 
Ribeiro
J
, 
Baracat
EC
, 
Abrao
MS
, 
Kho
RM
. Transvaginal ultrasound for the diagnosis of adenomyosis: systematic review and meta‐analysis. J Minim Invasive Gynecol.
2018;25(2):257–264. 10.1016/j.jmig.2017.08.653
288640447

Van den
T
, 
Dueholm
M
, 
Leone
FP
, 
Valentin
L
, 
Rasmussen
CK
, 
Votino
A
, et al. Terms, definitions and measurements to describe sonographic features of myometrium and uterine masses: a consensus opinion from the Morphological Uterus Sonographic Assessment (MUSA) group. Ultrasound Obstet Gynecol. 2015;46:284–298.2565268510.1002/uog.148068

Van den Bosch
T
, 
de Bruijn
AM
, 
de Leeuw
RA
, 
Dueholm
M
, 
Exacoustos
C
, 
Valentin
L
, et al. Sonographic classification and reporting system for diagnosing adenomyosis. Ultrasound Obstet Gynecol.
2019;53(5):576–582. 10.1002/uog.19096
297902179

Exacoustos
C
, 
Manganaro
L
, 
Zupi
E
. Imaging for the evaluation of endometriosis and adenomyosis. Best Pract Res Clinic Obstet Gynaecol.
2014;28:655–681.10.1016/j.bpobgyn.2014.04.0102486124710

Bazot
M
, 
Cortez
A
, 
Darai
E
, 
Rouger
J
, 
Chopier
J
, 
Antoine
JM
, et al. Ultrasonography compared with magnetic resonance imaging for the diagnosis of adenomyosis: correlation with histopathology. Hum Reprod. 2001;16:2427–2433.1167953310.1093/humrep/16.11.242711

Liu
X
, 
Ding
D
, 
Ren
Y
, 
Guo
SW
. Transvaginal elastosonography as an imaging technique for diagnosing adenomyosis. Reprod Sci. 2018;25:498–514.2932095610.1177/1933719117750752

### 3.3 CQ2: Are hormonal agents effective for adenomyosis‐associated pain?

#### 3.3.1 CQ 2‐1: How should adenomyosis‐associated pain be managed?

1. In general Medical therapy is effective in treating adenomyosis‐associated pain symptoms.

**Table:** 

Level of evidence	1b
Grade of recommendation	B

Systematic Reviews: 3

RCTs: 3

Cohort Studies: 1

##### Commentary

Dysmenorrhea is present in 50%−93% of patients with adenomyosis.^1^ We found three systematic reviews on the use of medications to treat adenomyosis‐associated pain.^1^, ^2^, ^3^ The efficiency of GnRH agonists, norethisterone, danazol, dienogest, LNG‐IUS (Mirena), OCs/LEPs, and NSAIDs, other agents under the investigation, such as aromatase inhibitors, selective progesterone receptor modulators, and GnRH antagonists have also been introduced. Few controlled trials have been conducted to provide evidence for the effects of the above agents, mostly from single‐arm interventional studies.

An OC/LEP (ethinyl estradiol 30 μg plus gestodene 75 μg for 21 days followed by a 7‐day withdrawal period) demonstrated a reduction in pain 6 months after use compared to baseline (visual analog scale [VAS]: 6.5 → 3.9). An LNG‐IUS showed a pain improvement similar to but superior to that of OCs/LEPs (VAS: 6.2 → 1.7).^4^ Following 12 weeks of oral letrozole (2.5 mg/day) or subcutaneous goserelin (3.6 mg/month), the improvement rates in pelvic pain and dysmenorrhea were 83.3% and 57.1%, respectively, in the letrozole group versus 92.8% and 100%, in the goserelin group; thus, the goserelin group demonstrated a more significant improvement in pelvic pain.^5^

In one RCT, dienogest and a placebo were compared in patients with adenomyosis in which patients with a hemoglobin level <8.0 g/dL or muscle layer thickness >4 cm were excluded.^6^ After 16 weeks, the dienogest group demonstrated significant reductions in the pain score, pain severity score, analgesic use score, and VAS. In another trial, dienogest (2 mg/day) and triptorelin (3.75 mg/4 weeks) were equally effective in reducing dyspareunia and chronic pelvic pain. At 16 weeks, triptorelin was more effective in improving dysmenorrhea (VAS: 30.6 vs. 0).^7^ There is an urgent need for randomized control tests (RCTs) to address the medical treatment of adenomyosis.

#### 3.3.2 CQ 2‐2: Are oral contraceptive/ low dose estrogen‐progestin (OCs/LEPs) effective for adenomyosis‐associated pain?

OCs/LEPs are effective in reducing adenomyosis‐associated pain.

**Table:** 

Level of evidence	1b
Grade of recommendation	B

Systematic Reviews	1
Cohort	1

##### Commentary

In a systematic review,^1^ the authors found only one study that compared COC with LNG‐IUS. The included COC to treat adenomyosis,^8^ containing 75 μg of gestodene + 30 μg of ethynylestradiol, was taken for 21 days with 7 days without the pills (21/7), compared to LNG‐IUS. The results showed a reduction of pain (6.55 ± 0.68–3.90 ± 0.54, *p* < 0.001) and a reduction in uterine volume, but it was still less efficient than LNG‐IUS for all evaluated parameters (pain: 6.23 ± 0.67–1.68 ± 1.25–*p* < 0.001).

#### 3.3.3 CQ 2‐3: Are GnRH agonists effective for adenomyosis‐associated pain?

GnRH agonists are effective in reducing endometriosis‐associated pain.

**Table:** 

Level of evidence	1b
Grade of recommendation	B

Systematic Reviews	1
Cohort	2

##### 3.3.3.1 Commentary

The health care burden of adenomyosis is substantial: 82.0% of women undergo hysterectomies, nearly 70% will have imaging studies suggestive of adenomyosis, and 37.6% are using chronic pain medications.^9^ GnRH agonists were the first drugs used in the treatment of adenomyosis, which resulted in a significant reduction of uterine size and a decrease in the severity of pain and abnormal bleeding symptoms,^10^ and since GnRH agonists are very popular in clinical practice for adenomyosis. However, due to the hypoestrogenic caused by GnRH analogs use, few side effects usually occur, including vasomotor syndrome, reduced bone mineral density, genital atrophy, and mood swings. Therefore, an add‐back therapy, either using estrogen and/or progestin been recommended to minimize side effects. Long‐term treatment with GnRH agonists should be restricted to women refractory to other medications or when surgery is contraindicated in high‐risk patients.^3^

We found one systematic review.^1^ Two other studies evaluated the use of GnRH agonists: one compared to an aromatase inhibitor (goserelin × letrozole)^5^ and the other to dienogest (triptorelin × dienogest).^7^ The GnRH agonist was more efficient than the aromatase inhibitor in controlling chronic pelvic pain (CPP) (*p* = 0.04), but they were equally efficient in managing dysmenorrhea and dyspareunia. Compared to dienogest, the GnRH analog was more efficient in controlling dysmenorrhea at 16 weeks (30.6 ± 18.4 vs. 0.0, *p* < 0.0001) but equally efficient at reducing dyspareunia and CPP. However, one study evaluated side effects and reported hot flushes as a major side effect in 81.3% of women treated with GnRH agonists.^5^

#### 3.3.4 CQ 2‐4: Are progestins effective for adenomyosis‐associated pain?

1. Dienogest is effective in reducing adenomyosis‐associated pain.

**Table:** 

Level of evidence:	1b
Grade of recommendation	B

2. Dienogest may delay the recurrence of adenomyosis‐associated symptoms up to 12 months after GnRH agonist use

**Table:** 

Level of evidence:	2b
Grade of recommendation	B

3. Treatment with dienogest may be associated with abnormal uterine bleeding in women with big uterine size

**Table:** 

Level of evidence:	2b
Grade of recommendation	B

4. Levonorgestrel intrauterine system (LNG‐IUS) is effective for adenomyosis‐associated pain

**Table:** 

Level of evidence:	Ib
Grade of recommendation	B

Systematic Reviews	2
RCT	1
Case‐Control Studies	6

##### Commentary

Progestins are known to trigger endometrial decidualization and cause endometrial atrophy. Consequently, they have long been known to be effective for endometriosis. However, they also trigger irregular vaginal bleeding as an adverse effect and are often combined with estrogen preparations. As a result, the use of progestins alone has become uncommon. Furthermore, due to the low level of progesterone receptor expression in endometriotic lesions, it is now considered necessary to take high doses of progestin to achieve a therapeutic effect.

#### 3.3.5 Dienogest (DNG)

DNG is an oral progestin made commercially available in Japan in January 2008, earlier than in any other country worldwide.^11^ DNG, a 19‐nortestosterone derivative, is an antiandrogenic drug with high selectivity for progesterone receptors (PRs) and has been used to treat adenomyosis. DNG suppresses ovarian function and proves highly effective in treating chronic pelvic pain.^6^ In addition, DNG directly inhibited cellular proliferation and induced apoptosis in human adenomyotic cells.^12^

In adenomyosis, three studies evaluated DNG; one compared to GnRH analog (triptorelin),^7^ the other to placebo,^6^ and one retrospective study evaluated the safety of DNG in women with adenomyosis.^13^ DNG was efficient in all three studies in reducing pain complaints (dysmenorrhea, dyspareunia, and CPP). When DNG was compared to the GnRH analog (triptorelin), both were similar to control dyspareunia (20.7 ± 16.5 vs. 25.8 ± 19.1, *p* = 0.3899) and CPP (21.7 ± 11.6 vs. 24.5 ± 13.8, *p* = 0.5076). There was a significant difference in the posttreatment dysmenorrhea between DNG and triptorelin at 16 weeks when the GnRH analog presented a better result (30.6 ± 18.4 vs. 0.0, *p* < 0.0001).^7^

Ono et al.^13^ reported that thirteen women continued DNG, and seven discontinued DNG within 12 months because of abnormal uterine bleeding. Moreover, the uterine size was significantly more prominent in the discontinuation group than in the continuation group. All uterine measurements had high Spearman rank correlation coefficients (*ρ* > 0.8, *p* < 0.05). In addition, adenomyosis was in the anterior wall in six cases in the discontinuation group. Measuring the uterus size and locating adenomyosis by TVUS are simple methods of predicting DNG discontinuation due to AUB.

Although many women reported bleeding control during DNG treatment, a quarter of them maintained bleeding versus none from the GnRH group.^7^ Similarly, uterine volume was reduced according to two studies in women who used DNG, but this reduction was lower than that obtained with the study that used GnRH analog (278 ± 162 vs. 151 ± 117 mL *p* = 0.01). One of these studies reported hot flushes (5.3%) as a side effect of the DNG.

One retrospective cohort study reported the recurrence of adenomyosis after GnRH agonist discontinuation. The study included 30 patients, divided into a group whose progress was observed without providing additional therapy following GnRHa administration for 6 months (Group G) and a group of patients administered DNG for 6 months following 6 months GnRHa administration (Group D). Abnormal uterine bleeding, dysmenorrhea, chronic pelvic pain, abdominal fullness, and uterine volume were recorded before treatment, 6 months after the start of therapy (6M), and 12 months after the beginning of treatment (12M). In Group G (*n* = 15), although all subjective symptoms disappeared at 6M, nearly all symptoms recurred at 12M. Uterine volume significantly decreased from 341.0 cm^3^ to 156.0 cm^3^ at 6M (*p* = 0.001) and significantly increased again to 282.3 cm^3^ at 12M (*p* = 0.003). In Group D (*n* = 15), all subjective symptoms disappeared at 6M, and only abdominal fullness returned in a significant number of patients (5 of 5; *p* = 0.021) at 12M. Uterine volume decreased significantly at 6M (*p* = 0.003) and significantly increased again from 162.5 to 205.6 cm^3^ at 12M (*p* = 0.006). Subjective symptoms, except for abdominal fullness, did not recur when the DNG was used after GnRHa.

DNG may not be good for patients with intrinsic and diffuse adenomyosis who complained of HMB.^14^, ^15^ DNG has been covered by the national insurance in Japan, but patients with large uterus (>10 cm) and anemia (Hb < 8 g/dL) are contraindicated.

An RCT enrolled 157 women with adenomyosis. Women were randomized to either LNG‐IUS (*n* = 76) or DNG (*n* = 81) groups as a controlled clinical trial for 72 months (6 years). LNG‐IUS and DNG both reduced pain scores in patients with adenomyosis. Concerning pain control, DNG offered greater efficacy than LNG‐IUS in 3 months of treatment.^15^

#### 3.3.6 Levonorgestrel intrauterine system (LNG‐IUS)

LNG‐IUS treatment is generally adequate for pain and heavy uterine bleeding, these effects can be due to (a) progestogenic effect on adenomyosis foci, (b) atrophy of the eutopic endometrium, and (c) control of endometrial factors that changed during adenomyosis.^15^ Two studies have assessed the use of LNG‐IUS versus hysterectomy or combined oral contraceptives (COC) in patients with adenomyosis.^8^, ^16^ In the first prospective randomized clinical trial, the LNG‐IUS effectively controlled bleeding, shown in the improvement of hemoglobin levels and reduced the number of days with bleeding. In the second study, a reduction in the number of days with bleeding was observed. In addition, there was an improvement in health‐related QOL variables.

Shabaan et al.^4^ assessed the pain scores. The LNG‐IUS was more efficient in the improvement of chronic pelvic pain than COC (6.23 ± 0.67 vs. 1.68 ± 1.25 – *p* < 0.001), as well as the reduction in uterine volume (10.23 ± 1.06 mL vs. 7.63 ± 0.49 mL, *p* < 0.001).

### 3.4 CQ 2‐5: GnRH antagonists for adenomyosis‐associated pain symptoms

At the moment, there is insufficient evidence to recommend the use of GnRH antagonists for adenomyosis‐associated pain symptoms.

Level of evidence: 4

Grade of recommendation: C

Case report: 2

There are two case reports on using GnRH antagonists to treat adenomyosis. Donnez et al.^17^ reported a case of a patient who was prescribed Linzagolix, a GnRH antagonist for adenomyosis, after failing a course of ulipristal acetate. Linzagolix significantly reduced adenomyotic lesion size and improved the patient's dysmenorrhea and quality of life. Similarly, Kavoussi et al.^18^ reported a case of a 41‐year‐old patient who presented with a fundal adenomyoma that regressed in size after treatment with improvement in her pelvic pain scores with Elagolix, another GnRH antagonist. These observations make it worth further looking into GnRH antagonists as a prospective treatment option for adenomyosis.

### 3.5 CQ2‐6: Medical treatment after adenomyomectomy

Level of evidence: 2b

Grade of recommendation: C

Number of studies referenced

RCT: 1

Cohort: 1

One RCT compared the efficacy of GnRH agonist and GnRH agonist + LNG‐IUS after adenomyomectomy for improved adenomyosis‐associated symptoms.^19^ In the 193 patients with adenomyosis, three groups were generated: adenomyomectomy (*n* = 57, group 1), adenomyomectomy + GnRHa (*n* = 83, group 2), and adenomyomectomy + GnRHa + LNG‐IUS (*n* = 53, group 3). The VAS scores of all patients reduced from 7.3 (6.0, 8.5) to 0 (0, 0.6) the 6 months after surgery, which was significantly higher in group 1 compared to other groups (*p* < 0.05). The dysmenorrhea recurrences were 26.3%, 6.1%, and 5.9% in groups 1, 2, and 3, respectively, at 36 months, which was significantly higher in group 1 (*p* < 0.01). Significantly decreased uterine volumes were observed in all patients from 222.2 (147.6, 350.4) to 77.0 (65.9, 94.1) mL (*p* < 0.05) at the 6 months after surgery.

In a retrospective study of 133 patients with symptomatic adenomyosis who underwent conservative uterine‐sparing surgery followed by gonadotropin‐releasing hormone agonist treatment, the intervention group (*n* = 54) immediately received a levonorgestrel‐releasing intrauterine system (LNG‐IUS) after surgery. Over a 12‐month follow‐up, the intervention group exhibited a more significant reduction in dysmenorrhea (mean ± SD: 6.5 ± 2.5 vs. 4.1 ± 3.6, *p* = 0.001). At the end of the 24‐month follow‐up period, the intervention group also exhibited a more significant reduction in dysmenorrhea as assessed with a VAS score (mean ± SD 6.1 ± 2.7 vs 3.7 ± 3.7, *p* = 0.002).^20^

Treatment of GnRH agonist and/or LNG‐IUS after conservative surgery for adenomyosis could significantly reduce the recurrence and pain scores.

### 3.6 CQ2‐7: Oral administration of norethisterone acetate (NETA) in women with adenomyosis

Norethisterone acetate (NETA) may be effective in women with adenomyosis.

**Table:** 

Level of evidence:	3b
Grade of recommendation	C

Cohort: 1

In a prospective cohort study (40 women),^21^ after 6 months of administration of NETA 5 mg daily, a significant decrease in each ultrasound feature of adenomyosis, the treatment significantly reduced uterine volume, size of focal adenomyosis foci, and in the intramyometrial cysts. In addition, after 6‐months of treatment, pain symptoms and AUB showed a significant improvement.

REFERENCES1

Benetti‐Pinto
CL
, 
Mira
TAA
, 
Yela
DA
, 
Teatin‐Juliato
CR
, 
Brito
LGO
. Pharmacological treatment for symptomatic adenomyosis: a systematic review. Rev Bras Ginecol Obstet. 2019;41:564–574.3154627810.1055/s-0039-16957372

Pontis
A
, 
D'alterio
M
, 
Pirarba
S
, 
De Angelis
C
, 
Tinelli
R
, 
Angioni
S
. Adenomyosis: a systematic review of medical treatment. Gynecol Endocrinol. 2016;32(9):696–700.2737997210.1080/09513590.2016.11972003

Vannuccini
S
, 
Luisi
S
, 
Tosti
C
, 
Sorbi
F
, 
Petraglia
F
. Role of medical therapy in the management of uterine adenomyosis. Fertil Steril. 2018;109(3):398–405.2956685210.1016/j.fertnstert.2018.01.0134

Shaaban
OM
, 
Ali
MK
, 
Sabra
AMA
, 
Abd El Aal
DEM
. Levonorgestrel‐releasing intrauterine system versus a low‐dose combined oral contraceptive for treatment of adenomyotic uteri: a randomized clinical trial. Contraception. 2015;92(4):301–307.2607167310.1016/j.contraception.2015.05.0155

Badawy
AM
, 
Elnashar
AM
, 
Mosbah
AA
. Aromatase inhibitors or gonadotropin‐releasing hormone agonists for the management of uterine adenomyosis: a randomized controlled trial. Acta Obstet Gynecol Scand. 2012;91(4):489–495.2222925610.1111/j.1600-0412.2012.01350.x6

Osuga
Y
, 
Fujimoto‐Okabe
H
, 
Hagino
A
. Evaluation of the efficacy and safety of dienogest in the treatment of painful symptoms in patients with adenomyosis: a randomized, double‐blind, multicenter, placebo‐controlled study. Fertil Steril. 2017;108(4):673–678.2891193410.1016/j.fertnstert.2017.07.0217

Fawzy
M
, 
Mesbah
Y
. Comparison of dienogest versus triptorelin acetate in premenopausal women with adenomyosis: a prospective clinical trial. Arch Gynecol Obstet. 2015;292(6):1267–1271.2599048010.1007/s00404-015-3755-58

Shaaban
OM
, 
Ali
MK
, 
Sabra
AM
, 
Abd El Aal
DE
. Levonorgestrel‐releasing intrauterine system versus a low‐dose combined oral contraceptive for treatment of adenomyotic uteri: a randomized clinical trial. Contraception. 2015;92(4):301–307.2607167310.1016/j.contraception.2015.05.0159

Yu
O
, 
Schulze‐Rath
R
, 
Grafton
J
, 
Hansen
K
, 
Scholes
D
, 
Reed
SD
. Adenomyosis incidence, prevalence and treatment: United States population‐based study 2006‐2015. Am J Obstet Gynecol.
2020;223:94.e1–94.e10.10.1016/j.ajog.2020.01.0163195415610

Grow
D
, 
Filer
R
. Treatment of adenomyosis with long‐term GnRH analogues: a case report. Obstet Gynecol.
1991;78(3 Pt 2):538–539.190806911

Harada
T
, 
Momoeda
M
, 
Taketani
Y
, 
Aso
T
, 
Fukunaga
M
, 
Hagino
H
, et al. Dienogest is as effective as intranasal buserelin acetate for the relief of pain symptoms associated with endometriosis—a randomized, double‐blind, multicenter, controlled trial. Fertil Steril.
2008;91(3):675–681.1865318410.1016/j.fertnstert.2007.12.08012

Yamanaka
A
, 
Kimura
F
, 
Kishi
Y
, 
Takahashi
K
, 
Suginami
H
, 
Shimizu
Y
, et al. Progesterone and synthetic progestin, dienogest, induce apoptosis of human primary cultures of adenomyotic stromal cells. Eur J Obstet Gynecol Reprod Biol. 2014;179:170–174.2495382210.1016/j.ejogrb.2014.05.03113

Ono
N
, 
Asano
R
, 
Nagai
K
, 
Sugo
Y
, 
Nakamura
T
, 
Miyagi
E
. Evaluating the safety of dienogest in women with adenomyosis: a retrospective analysis. J Obstet Gynaecol Res. 2021;47(4):1433–1440.3359065610.1111/jog.1461214

Matsubara
S
, 
Kawaguchi
R
, 
Akinishi
M
, 
Nagayasu
M
, 
Iwai
K
, 
Niiro
E
, et al. Subtype I (intrinsic) adenomyosis is an independent risk factor for dienogest‐related serious unpredictable bleeding in patients with symptomatic adenomyosis. Sci Rep. 2019;9(1):17654.3177640410.1038/s41598-019-54096-zPMC688134415

Ota
I
, 
Taniguchi
F
, 
Ota
Y
, 
Nagata
H
, 
Wada
I
, 
Nakaso
T
, et al. A controlled clinical trial comparing potent progestins, LNG‐IUS and dienogest, for the treatment of women with adenomyosis. Reprod Med Biol. 2021;20(4):427–434.3464607010.1002/rmb2.12408PMC849960316

Ozdegirmenci
O
, 
Kayikcioglu
F
, 
Akgul
MA
, 
Kaplan
M
, 
Karcaaltincaba
M
, 
Haberal
A
, et al. Comparison of levonorgestrel intrauterine system versus hysterectomy on efficacy and quality of life in patients with adenomyosis. Fertil Steril. 2011;95(2):497–502.2107415010.1016/j.fertnstert.2010.10.00917

Donnez
O
, 
Donnez
J
. Gonadotropin‐releasing hormone antagonist (linzagolix): a new therapy for uterine adenomyosis. Fertil Steril. 2020;114(3):640–645.3250731510.1016/j.fertnstert.2020.04.01718

Kavoussi
SK
, 
Esqueda
AS
, 
Jukes
LM
. Elagolix to medically treat a uterine adenomyoma: a case report. Eur J Obstet Gynecol Reprod Biol. 2020;247:266–267.3208807010.1016/j.ejogrb.2020.02.02719

Li
Q
, 
Yuan
M
, 
Li
N
, 
Zhen
Q
, 
Chen
C
, 
Wang
G
. The efficacy of medical treatment for adenomyosis after adenomyomectomy. J Obstet Gynaecol Res. 2020;46(10):2092–2099.3272568210.1111/jog.1437620

Lin
CJ
, 
Hsu
TF
, 
Chang
YH
, 
Huang
BS
, 
Jiang
LY
, 
Wang
PH
, et al. Postoperative maintenance levonorgestrel‐releasing intrauterine system for symptomatic uterine adenomyoma. Taiwan J Obstet Gynecol.
2018;57(1):47–51.2945890210.1016/j.tjog.2017.12.03621

Lazzeri
L
, 
Exacoustos
C
, 
Monti
G
, 
Centini
G
, 
Conway
F
, 
Barbanti
C
, et al. OC08.04: Oral administration of norethisterone acetate in women with adenomyosis: effects on ultrasound features and clinical symptoms. Ultrasound Obstet Gynecol. 2019;54:20.

## 4 CQ3: IS SURGICAL REMOVAL EFFECTIVE FOR ADENOMYOSIS‐ASSOCIATED PAIN?

1. Uterine‐sparing surgery reduces dysmenorrhea

**Table:** 

Level of evidence:	1b
Grade of recommendation:	B

2. There is no difference between laparoscopic and laparotomy approaches in pain reduction

**Table:** 

Level of evidence:	2b
Grade of recommendation:	C

3. Hysterectomy reduces adenomyosis associated pain

**Table:** 

Level of evidence:	1b
Grade of recommendation:	B

Meta‐analysis	3
Systematic Reviews	6
Clinical trial	3
Descriptive Study	4

### 4.1 Commentary

In practice, surgical treatment is recommended in cases of ineffective conservative therapy and severe forms of adenomyosis (diffuse and focal). A rare form of focal adenomyosis is described in the literature by various terms‐ juvenile cystic adenomyosis (JCA), cystic myometrial lesions, accessory uterine cavity masses, or juvenile adenomyotic cysts. The latter is best treated surgically. Reliable data about the correlation between pain and postoperative outcomes are not described.^1^

In general, all surgical methods can be divided into two groups: nonexcisional techniques such as thermal coagulation of the diseased myometrium, and excisional techniques including hysteroscopic resection for JCA,^1^, ^2^ adenomyomectomy with complete removal of the focal disease, usually adenomyoma, and myometrectomy, with partial removal of the diseased myometrium, usually diffuse type. In case of the impossibility of organ‐preserving interventions, hysterectomy is indicated.

We have analyzed how organ‐preserving surgery affects the reduction of pain syndrome in adenomyosis. Most studies show that uterine‐sparing surgery reduces dysmenorrhea: after complete excision 50%–94.7% and after incomplete excision 41%–94%.^3^, ^4^, ^5^ According to the study by Zhu et al.,^6^ when comparing laparoscopic and laparotomic approaches (double‐flap adenomyomectomy), no difference was found (75% and 62,1% in pain relief, respectively) and efficacy was maintained after 6 years of follow‐up more than 60% of patients that show the possibility of recurrence of symptoms occurring in 9% of patients in complete excision group and 19% of patient in partial excision group.^7^

If symptoms persist despite ongoing medical or other types of conservative therapy, the final preference is given to a radical method of surgical treatment – hysterectomy. According to the results of the meta‐analysis, the pain after a hysterectomy is reduced by 84%.^5^ But several studies have reported persistent pelvic pain after a hysterectomy for adenomyosis.^8^

A nonsystematic review showed 23 cases of uterine rupture out of 2365 women who underwent adenomyomectomy (1.0%).^9^ The author concluded that uterine rupture after uterus‐sparing surgical treatment of adenomyosis seems to be related to the removal of adenomyotic tissue technique, the degree of remnants of adenomyosis left postoperatively, the uterine wall, postoperative complications, and the interval between the procedure and conception. Cesarean section is a preferred delivery route after adenomyosis excision treatment.

REFERENCES1

Kerbage
Y
, 
Dericquebourg
S
, 
Collinet
P
, 
Verpillat
P
, 
Giraudet
G
, 
Rubod
C
. Cystic adenomyoma surgery. J Gynecol Obstet Hum Reprod. 2022;51(3):102313. 10.1016/j.jogoh.2022.102313
350315102

Di Spiezio
SA
, 
Calagna
G
, 
Santangelo
F
, 
Zizolfi
B
, 
Tanos
V
, 
Perino
A
, et al. The role of hysteroscopy in the diagnosis and treatment of adenomyosis. BioMed Res Intl.
2017;2017:2518396. 10.1155/2017/2518396
PMC5568620288526463

Younes
G
, 
Tulandi
T
. Conservative surgery for adenomyosis and results: a systematic review. J Minim Invasive Gynecol. 2018;25(2):265–276. 10.1016/j.jmig.2017.07.014
287394144

Oliveira
MAP
, 
Crispi
CP
Jr
, 
Brollo
LS
, 
Crispi
CP
, 
De Wilde
RL
. Surgery in adenomyosis. Arch Gynecol Obstet. 2018;297(3):581–589. 10.1007/s00404-017-4603-6
291979875

Mikos
T
, 
Lioupis
M
, 
Anthoulakis
C
, 
Grimbizis
GF
. The outcome of fertility‐sparing and nonfertility‐sparing surgery for the treatment of adenomyosis. A systematic review and meta‐analysis. J Minim Invasive Gynecol. 2020;27(2):309–331. 10.1016/j.jmig.2019.08.004
313984156

Zhu
L
, 
Chen
S
, 
Che
X
, 
Xu
P
, 
Huang
X
, 
Zhang
X
. Comparisons of the efficacy and recurrence of adenomyomectomy for severe uterine diffuse adenomyosis via laparotomy versus laparoscopy: a long‐term result in a single institution. J Pain Res. 2019;12:1917–1924. 10.2147/JPR.S205561
31303783PMC66032877

Sharara
FI
, 
Kheil
MH
, 
Feki
A
, 
Rahman
S
, 
Klebanoff
JS
, 
Ayoubi
JM
, et al. Current and prospective treatment of adenomyosis. J Clin Med. 2021;10(15):3410. 10.3390/jcm10153410
34362193PMC83481358

Li
JJ
, 
Chung
JPW
, 
Wang
S
, 
Li
TC
, 
Duan
H
. The investigation and management of adenomyosis in women who wish to improve or preserve fertility. BioMed Res Int.
2018;2018:6832685. 10.1155/2018/6832685
29736395PMC58750649

Osada
H
. Uterine adenomyosis and adenomyoma: the surgical approach. Fertil Steril. 2018;109:406–417.2956685310.1016/j.fertnstert.2018.01.032

## 5 CQ4: ARE NONSURGICAL TREATMENTS EFFECTIVE FOR ADENOMYOSIS‐ASSOCIATED PAIN?

1. Uterine artery embolization is an effective nonsurgical treatment option for the treatment of adenomyosis

**Table:** 

Level of evidence	2a
Grade of recommendation	B

2. High‐frequency ultrasound is a safe and effective nonsurgical treatment option for the treatment of adenomyosis.

**Table:** 

Level of evidence	2a
Grade of recommendation	B

3. Thermal ablation, including radiofrequency ablation or microwave ablation, is a safe and effective nonsurgical treatment option for the treatment of adenomyosis.

**Table:** 

Level of evidence	3b
Grade of recommendation	C

Practice Guidelines: 2

Systematic Review/Meta‐analysis: 3

RCTs: 2

Non‐RCT: 4

Clinical Trial/Cohort Study: 4

Opinion of expert panel: 4

### 5.1 Commentary

In the past two decades, minimally invasive nonsurgical uterus‐sparing techniques have been used for treating adenomyosis as an alternative to hysterectomy.^1^ These techniques work by causing necrosis of the adenomyotic tissue thereby decreasing symptoms including dysmenorrhea and menorrhagia. Uterine artery embolization (UAE) causes avascular necrosis of the tissue whereas High‐Frequency Ultrasound (HIFU) and thermal ablation cause thermal necrosis. All techniques are well known and have been used for treating other gynecological indications including fibroids and dysfunctional uterine bleeding.

### 5.2 Uterine artery embolization (UAE)

#### 5.2.1 Technique

Uterine artery embolization is the use of transarterial catheters through the femoral or radial artery under fluoroscopic guidance for injecting permanent particles to selectively block the uterine artery bilaterally. The aim is to induce more than 34% necrosis within adenomyotic tissues.^2^ Care should be taken to avoid blocking the cervicovaginal and ovarian artery branches.^3^

#### 5.2.2 Success rates

Overall improvement of symptoms is 83.1% with patients treated for combined adenomyosis with fibroids showing better results than patients with pure adenomyosis.^4^ Success rates also depend on the vascularity of adenomyosis with hypervascular lesions showing higher long‐term success rates than hypovascular lesions (83.6% vs. 52.8%).^5^

#### 5.2.3 Complications

Adverse events related to UAE for adenomyosis include postprocedure short‐term pain (87%), persistent amenorrhea (6%–21%) in patients over 40, and the need for hysterectomy in the long term (14%).^6^ Control of necrosis with UAE is a problem and sometimes more than 90% of the myometrium can become necrotic.^1^ Current American College of Obstetrics and Gynecology and Society of Interventional Radiology guidelines still consider the desire for future fertility a relative contraindication to UAE.^6^, ^7^ As there are no completed randomized controlled trials on UAE and adenomyosis, an ongoing trial named QUESTA (QUality of life after Embolization vs. hySTerectomy in Adenomyosis) should eventually give more accurate information about the success and complication rates of UAE.^8^

### 5.3 High‐frequency ultrasound

#### 5.3.1 Technique

High‐intensity focused ultrasound (HIFU) therapy for the management of adenomyosis induces focal thermocoagulation of the adenomyotic lesions. The treatment may be targeted and monitored by ultrasound or MRI. The major shortcoming is technical eligibility, and 60% of women eligible for treatment for fibroids by UAE were not eligible for treatment by MRgFUS.^9^ The treatment can only be recommended to symptomatic premenopausal women with adenomyosis and no plans for future pregnancy, no suspected pelvic adhesions, no lower abdominal surgery, body weight less than 100 kg, and abdominal wall thickness less than 5 cm. Lesions in adenomyosis that can be treated have a diameter of 3–10 cm.^10^

Ultrasound contrast agents (microbubbles) and hormonal (GnRH) and nonhormonal (metformin) treatments may reduce the recurrence risk and enhance the efficacy of HIFU. Microbubbles are believed to improve the ablative effects of HIFU by changing the acoustic characteristics, thus increasing energy deposition in target tissues, while GnRH and metformin inhibit cellular proliferation and induce apoptosis.^3^

#### 5.3.2 Success rates

Follow‐up of more than one year with the evaluation of symptoms has been accomplished in 6 trials.^1^ A sustained effect with symptom relief after 12 months was obtained in 88% of 669 women treated. Other effects were a reduction in symptom severity score (SSS) questionnaire score of 25%–65%, increased UFS‐QOL from 39% to 85%, decreased dysmenorrhea, and a reduction in a mean uterine volume of 22%–54%. However, most published studies are retrospective and few, if any, adopted randomized clinical trial format. Therefore, caution should be exercised when reading published studies.

#### 5.3.3 Complications

The coagulation necrosis obtained with HIFU is much less painful than that obtained by UAE.^1^ Safety has been evaluated 2549 women had adenomyosis.^11^ The most common adverse effect was vaginal secretion (9%) for less than 3 weeks and lower abdominal pain, which lasted more than 24 h (2%). Only 0.6% had serious complications and no permanent injury or fatal complication occurred.

### 5.4 Thermal ablation

#### 5.4.1 Technique

Thermal ablation can be performed by needle application or global ablation. Needle application can be performed by transcervical, percutaneous, or laparoscopy. Global ablation is performed within the uterine cavity by vaginal approach. Needle applications can use radiofrequency ablation (RFA) or microwave ablation (MWA). In radiofrequency ablation (RFA), a high‐frequency (450–500 kHz) alternating electrical current is used to create ionic agitation, which produces frictional heat and heat conduction to achieve subsequent tissue necrosis.^12^ RFA's mechanism of heating necessitates slow heating to 50–100°C to avoid charring and vaporization, which could compromise electrical current flow.^13^ Microwave ablation (MWA) uses electromagnetic energy (915 MHz–2.45 GHz) to rapidly rotate adjacent polar water molecules and heat tissue to lethal temperatures greater than 150°C.^14^ It works by dielectric hysteresis, which is a process in which polar molecules, primarily water are forced to continuously realign with the oscillating electric field generating kinetic energy and subsequent heat generation. MWA has faster ablation times, larger ablation volumes, effectiveness in many tissue types, applications, and an improved convection profile when compared to RFA.^14^

#### 5.4.2 Success rates

Laparoscopic RFA (LRFA) has shown promising results in a retrospective cohort study with an 87% reduction in the need for hysterectomy.^15^ A significant reduction was also seen in VAS scores for all components including, dysmenorrhea, dyspareunia, dyschezia, dysuria, and chronic pelvic pain over long‐term follow‐up. USgRFA postprocedure pregnancy rates are 50% with a 60% cesarean section rate and a 33.3% rate of spontaneous abortions.^16^ No uterine ruptures occurred. A review on MWA showed postprocedure uterine volume reduction rate was 55.2%–64.9%, and the adenomyosis volume reduction rate was 64.9%–93.1% after 12 months of follow‐up.^17^ After treatment, patients’ clinical symptoms significantly improved with dysmenorrhea decreasing by 50%–81.7%, anemia reduction rates of 55.6%–78.5%, and an overall symptom severity score (SSS) questionnaire improvement by 20.9%–60.2%.^17^ In a comparative interventional study, the safety and success rates of Percutaneous MWA ultrasound‐guided RFA (USgRFA) in the treatment of uterine adenomyosis were similar.^12^ Dysmenorrhea was partial to completely resolved, respectively, in 95.0% and 92.4% of women at the end of the first year. However, the mean ablation time of PMWA was shorter than that of USgRFA.

#### 5.4.3 Complications

The common adverse events after both RFA and MWA treatments were abdominal pain, vaginal discharge, and low‐grade fever.^12^ The incidence rates of pain in the lower abdomen, at the puncture site, and the ablation site ranged from 43.9% to 100%, and the incidence rate of vaginal discharge ranged from 7.1% to 88.2%.^17^ Following MWA, Abdominal pain is self‐limited and lasts for no more than 14 days.^15^ Vaginal discharge usually lasts 2–11 days^18^ but in 22% of cases can last over a year.^19^ It is mainly caused by liquefaction necrosis and can be pink, light red, yellow, and brown. Long‐term complications such as infertility and damage to adjacent organs are uncommon and the recurrence rate of adenomyosis depends on the kind of lesion with a recurrence of 18% after 1–2 years for localized lesions versus 38% for diffuse adenomyosis.^17^ LRFA has shown a 20% postprocedure amenorrhea rate related to ovarian inactivity in patients aged over 40 years and a 6% postprocedure Asherman's Syndrome rate.^15^

REFERENCES1

Dueholm
M
. Minimally invasive treatment of adenomyosis. Best Pract Res Clin Obstet Gynaecol.
2018;51:119–137. 10.1016/j.bpobgyn.2018.01.016
295553802

Kim
MD
, 
Kim
YM
, 
Kim
HC
, 
Cho
JH
, 
Kang
HG
, 
Lee
C
, et al. Uterine artery embolization for symptomatic adenomyosis: a new technical development of the 1‐2‐3 protocol and predictive factors of MR imaging affecting outcomes. J Vasc Interv Radiol.
2011;22(4):497–502. 10.1016/j.jvir.2011.01.426
213778973

Dessouky
R
, 
Gamil
SA
, 
Nada
MG
, 
Mousa
R
, 
Libda
Y
. Management of uterine adenomyosis: current trends and uterine artery embolization as a potential alternative to hysterectomy. Insights Imaging. 2019;10(1):48. 10.1186/s13244-019-0732-8
31030317PMC64869324

de Bruijn
AM
, 
Smink
M
, 
Lohle
PNM
, 
Huirne
JAF
, 
Twisk
JWR
, 
Wong
C
, et al. Uterine artery embolization for the treatment of adenomyosis: a systematic review and meta‐analysis. J Vasc Interv Radiol.
2017;28(12):1629–1642.e1. 10.1016/j.jvir.2017.07.034
290329465

Zhou
J
, 
He
L
, 
Liu
P
, 
Duan
H
, 
Zhang
H
, 
Li
W
, et al. Outcomes in adenomyosis treated with uterine artery embolization are associated with lesion vascularity: a long‐term follow‐up study of 252 cases. PLoS One.
2016;11(11):e0165610. 10.1371/journal.pone.0165610
27806072PMC50917596

Caridi
TM
, 
De la Garza‐Ramos
C
, 
Brook
OR
, 
Learman
LA
, 
Opoku‐Anane
J
, 
Phipps
D
, et al. Uterine artery embolization for symptomatic adenomyosis: Proceedings from a Society of Interventional Radiology Foundation Research Consensus Panel. J Vasc Interv Radiol.
2022;33(5):586–592. 10.1016/j.jvir.2022.01.017
354897887
American College of Obstetricians and Gynecologists’ Committee on Practice Bulletins‐Gynecology
. Management of symptomatic uterine leiomyomas: ACOG Practice Bulletin, Number 228. Obstet Gynecol. 2021;137(6):e100–e115. 10.1097/AOG.0000000000004401
340118888

de Bruijn
AM
, 
Lohle
PN
, 
Huirne
JA
, 
de Vries
J
, 
Twisk
M
, 
Group
QUESTA‐T
, et al. Uterine artery embolization versus hysterectomy in the treatment of symptomatic adenomyosis: protocol for the randomized QUESTA trial. JMIR Res Protoc.
2018;7(3):e47. 10.2196/resprot.8512
29496654PMC58569349

Froling
V
, 
Kroncke
TJ
, 
Schreiter
NF
, 
Scheurig‐Muenkler
C
, 
Collettini
F
, 
Hamm
B
, et al. Technical eligibility for treatment of magnetic resonance‐guided focused ultrasound surgery. Cardiovasc Intervent Radiol.
2014;37(2):445–450. 10.1007/s00270-013-0678-z
2383900510

Cheung
VY
. Current status of high‐intensity focused ultrasound for the management of uterine adenomyosis. Ultrasonography.
2017;36(2):95–102. 10.14366/usg.16040
28145109PMC538184511

Chen
J
, 
Chen
W
, 
Zhang
L
, 
Li
K
, 
Peng
S
, 
He
M
, et al. Safety of ultrasound‐guided ultrasound ablation for uterine fibroids and adenomyosis: a review of 9988 cases. Ultrason Sonochem.
2015;27:671–676. 10.1016/j.ultsonch.2015.05.031
2609367812

Lin
XL
, 
Hai
N
, 
Zhang
J
, 
Han
ZY
, 
Yu
J
, 
Liu
FY
, et al. Comparison between microwave ablation and radiofrequency ablation for treating symptomatic uterine adenomyosis. Int J Hyperthermia. 2020;37(1):151–156. 10.1080/02656736.2019.1708481
3202440213

Hong
K
, 
Georgiades
C
. Radiofrequency ablation: mechanism of action and devices. J Vasc Interv Radiol.
2010;21(8 Suppl):S179–S186. 10.1016/j.jvir.2010.04.008
2065622714

Kim
C.

Understanding the nuances of microwave ablation for more accurate post‐treatment assessment. Future Oncol.
2018;14(17):1755‐1764. doi: 10.2217/fon-2017-0736
2944181315

Stepniewska
AK
, 
Baggio
S
, 
Clarizia
R
, 
Bruni
F
, 
Roviglione
G
, 
Ceccarello
M
, et al. Heat can treat: long‐term follow‐up results after uterine‐sparing treatment of adenomyosis with radiofrequency thermal ablation in 60 hysterectomy candidate patients. Surg Endosc.
2022;36(8):5803‐5811. doi: 10.1007/s00464-021-08984-z
3502493016

Nam
JH
. Pregnancy and symptomatic relief following ultrasound‐guided transvaginal radiofrequency ablation in patients with adenomyosis. J Obstet Gynaecol Res.
2020;46(1):124‐132. doi: 10.1111/jog.14145
3164673117

Zhang
S
, 
Wang
K
, 
Di
A
, 
Yu
D
, 
Yao
T
. Ultrasound‐guided percutaneous microwave ablation of adenomyosis: a narrative review. Ann Palliat Med.
2021;10(11):12003–12011. 10.21037/apm-21-3133
3487232318

Liu
JX
, 
Li
JY
, 
Zhao
XY
, 
Zhang
QH
, 
Cao
Y
, 
Huang
XJ
, et al. Transvaginal ultrasound‐ and laparoscopy‐guided percutaneous microwave ablation for adenomyosis: preliminary results. Int J Hyperthermia.
2019;36(1):1233–1238. 10.1080/02656736.2019.1690169
3181816319

Xu
RF
, 
Zhang
J
, 
Han
ZY
, 
Zhang
BS
, 
Liu
H
, 
Li
XM
, et al. Variables associated with vaginal discharge after ultrasound‐guided percutaneous microwave ablation for adenomyosis. Int J Hyperthermia.
2016;32(5):504–510. 10.3109/02656736.2016.1150523
27087631

## 6 CQ5: IS SURGICAL REMOVAL EFFECTIVE FOR ADENOMYOSIS‐ASSOCIATED INFERTILITY?

Surgical removal of adenomyosis may be effective for adenomyosis‐associated infertility.

**Table:** 

Level of evidence	2b
Grade of recommendation	C

Retrospective cohort study 8

Systematic review (Nonconsecutive cohort study or limited population) 4

Nonconsecutive cohort study 6

Case series study 6

### 6.1 Commentary

Adenomyosis represents a common gynecological disorder with a negative impact on fertility. However, it remains challenging to establish if adenomyosis is the only cause of infertility or not. This disease is often associated with various gynecological pathologies especially with endometriosis but also with fibroids, polyps, and others. Therefore, patients may be affected by one or more of these concomitant diseases. This leads to significant bias when analyzing the effect of the treatment. The coexistence of endometriosis and adenomyosis avoids the impact of adenomyosis surgery per se on fertility.^1^ In addition, there are no universally accepted standardized diagnostic criteria for adenomyosis. This lack of a common consensus makes it difficult to evaluate treatment outcomes and compare studies where different criteria are used.

The answer to the question “Is surgical removal effective for adenomyosis‐associated infertility?” would be probably “yes, surgical removal of adenomyosis may be effective for adenomyosis‐associated infertility”, yet there is no hard scientific data in the medical literature.^2^, ^3^ Data regarding the impact of uterine‐sparing surgery (with medical therapy, e.g. GnRH agonists) on fertility potential is controversial because most of the published studies were not designed to address this issue. Furthermore, there are no controlled trials in the literature.

Prospectively, 103 Iranian patients with documented severe adenomyosis were candidates for adenomyomectomy over 7 years. Of 103 patients, 55.3% presented with infertility, 16.5% with IVF failure, and 8.7% with recurrent abortion. Of 70 patients who attempted pregnancy either naturally (*n* = 21) or using ART (*n* = 49), 30% became pregnant, and 16 pregnancies reached full term.

In a retrospective study, 53 patients who underwent conservative surgery (modified adenomyomectomy and wedge resection) were analyzed. In total, 33.3% of the patients with infertility in the modified adenomyomectomy group became pregnant. The authors recommended the infertile patients to be transferred directly to an ART center soon after adenomyomectomy.^4^ In the patients with diffuse adenomyosis, the rate of pregnancy (including ART) after laparoscopic surgery was low.^5^ The clinical pregnancy rate was a total of 31.4% among 102 women who desired to preserve their fertility after adenomyomectomy. When the women were divided into less than 39 and more than 40 years, clinical pregnancy rates were 41.3% and 3.7%, respectively. When fertility outcomes on women who had a history of IVF failures were analyzed, clinical pregnancy rates were 60.8% in the younger group and 7.1% in the older group.^6^

REFERENCES1

Shi
J
, 
Dai
Y
, 
Zhang
J
, 
Li
X
, 
Jia
S
, 
Leng
J
. Pregnancy outcomes in women with infertility and coexisting endometriosis and adenomyosis after laparoscopic surgery: a long‐term retrospective follow‐up study. BMC Pregnancy Childbirth. 2021;21(1):383. 10.1186/s12884-021-03851-0
34006232PMC81324062

Younes
G
, 
Tulandi
T
. Conservative surgery for adenomyosis and results: a systematic review. J Minim Invasive Gynecol. 2018;25(2):265–276. 10.1016/j.jmig.2017.07.014
287394143

Harada
T
, 
Khine
YM
, 
Kaponis
A
, 
Nikellis
T
, 
Decavalas
G
, 
Taniguchi
F
. The impact of adenomyosis on women's fertility. Obstet Gynecol Surv.
2016;71(9):557–568.2764061010.1097/OGX.0000000000000346PMC50499764

Sun
AJ
, 
Luo
M
, 
Wang
W
, 
Chen
R
, 
Lang
JH
. Characteristics and efficacy of modified adenomyomectomy in the treatment of uterine adenomyoma. Chin Med J.
2011;124:1322–1326.217407415

Nishida
M
, 
Takano
K
, 
Arai
Y
, 
Ozone
H
, 
Ichikawa
R
. Conservative surgical management for diffuse uterine adenomyosis. Fertil Steril.
2010;94:715–719.1940639610.1016/j.fertnstert.2009.03.0466

Kishi
Y
, 
Yabuta
M
, 
Taniguchi
F
. Who will benefit from uterus‐sparing surgery in adenomyosis‐associated subfertility?
Fertil Steril.
2014;102(3):802–807.2495477410.1016/j.fertnstert.2014.05.028

## 7 CQ6: ARE HORMONAL AGENTS EFFECTIVE FOR ADENOMYOSIS‐ASSOCIATED INFERTILITY?

1. It is not recommended to prescribe hormonal agents to increase the spontaneous pregnancy rate in women with adenomyosis.

Level of Evidence: 4

Grade of Recommendation: C

2. In specific situations, hormonal agents can be prescribed to improve in vitro fertilization (IVF) outcomes in women with adenomyosis.

2‐1) It is not recommended to administrate gonadotropin‐releasing hormone agonists (GnRHa) before fresh embryo transfer (ET) cycles to improve pregnancy outcomes.

Level of Evidence: 2b

Grade of Recommendation: B

2‐2) GnRHa administration before frozen embryo transfer (FET) may improve the pregnancy rate.

Level of Evidence: 2b

Grade of Recommendation: B

Meta‐analysis 2

Cohorts 8

Case Reports 1

Commentary

Adenomyosis is one of the causes of infertility and is known to increase the risk of miscarriage or premature birth.^1^ Besides, infertile women with adenomyosis represent lower assisted reproductive technology (ART) outcomes,^2^, ^3^ and several mechanisms have been proposed. (1) Irregular and excessive contractions of the uterus accompanying adenomyosis may interfere with fertilization by interfering with the movement of gametes and may affect implantation after embryo transfer. (2) The decidualization of the endometrium may also be adversely affected, and the implantation probability may be interfered.^4^ (3) Adenomyosis may cause altered endometrial‐myometrial vascular growth^5^. (4) Chronic inflammation accompanying adenomyosis affects the environment in the pelvis and uterus, which can adversely affect gametes, embryos, endometrium, and the implantation process.^6^, ^7^

Therefore, it can be inferred that by alleviating adenomyosis in infertile women accompanied by this disease, the results of infertility treatment including the pregnancy rate can be improved, and studies are being reported to verify this possibility. Gonadotropin‐releasing hormone agonist (GnRHa) has been attempted to control adenomyosis in infertile women wishing to become pregnant.^8^ GnRHa is known to exhibit antiproliferative and anti‐inflammatory effects in addition to hypoestrogenic effects.^9^, ^10^ In a prospective observational study, Xie et al. administered Triptorelin 3.75 mg every 28 days for 6 months to infertile women diagnosed with adenomyosis. As a result, 12 of 45 (26.7%) women became pregnant, and it was reported that the elasticity of adenomyosis measured by elastography significantly increased after GnRHa treatment in the pregnant group.^11^ This report suggests that GnRHa enhances spontaneous pregnancy by alleviating the adverse effects caused by adenomyosis, but this study has the limitation that there is no control group. Until now, there is no firm evidence through appropriate studies to prescribe hormonal agents for improving spontaneous pregnancy rates in women with adenomyosis.

Present studies on the effects of GnRHa pretreatment on artificial reproductive technologies (ART) results in infertile women with adenomyosis are summarized in Table 1. When reviewing the results of ultra‐long GnRHa protocol or GnRHa pretreatment in performing fresh embryo transfer (ET) in women with adenomyosis, some studies have reported that the live birth rate (LBR) was improved.^12^, ^13^ Lan et al. reported that LBR increased when only diffuse‐type adenomyosis cases were selectively analyzed, suggesting that the characteristics of adenomyosis may affect pregnancy outcomes. On the contrary, Chen et al. reported that the LBR was lowered when GnRHa pretreatment was performed.^14^ Other studies reported no difference in pregnancy outcomes or miscarriage rate.^15^, ^16^ Reviewing these results, in the case of fresh ET the effect of GnRHa pretreatment is still unclear, and it cannot be recommended to administrate GnRHa before fresh ET cycles to improve pregnancy outcomes.

Four studies have been published so far to analyze the effect of GnRHa pretreatment before frozen ET (FET) cycles in infertile women with adenomyosis. The most recent study by Wu et al. reported that FET after long‐term GnRHa pretreatment had a better clinical pregnancy rate (CPR) (59.3%) than fresh ET with long GnRHa protocol (43.5%), but no difference with fresh ET using ultra‐long GnRHa protocol (53.6%).^16^ Park et al. reported a higher, although not statistically different, CPR in patients with FET following GnRHa pretreatment (39.5%) compared to those who have undergone fresh ET without GnRHa pretreatment (25.2%) or fresh ET with GnRHa pretreatment (30.5%).^15^ Niu et al. analyzed only FET cycles and reported that CPR was improved with GnRHa pretreatment (51.4% vs. 24.8%).^17^ Unlike these three studies, Li et al. reported that there was no difference in CPR and LBR between FET cycles with and without GnRHa pretreatment.^18^ Therefore, in the cases of FET, GnRHa pretreatment may have a positive effect on pregnancy outcomes, and at least there is no evidence to date that this treatment has detrimental effects on pregnancy.

Since all these study results have the disadvantage of being a retrospective cohort study, it should be considered that selection bias may play a critical role. Moreover, it should also be noted that important variables such as the duration of GnRHa administration vary widely between studies and even within the same study.

Cozzolino et al. recently reported the results of a meta‐analysis on whether GnRHa pretreatment before IVF for infertile women with adenomyosis is effective to improve pregnancy outcomes.^3^ This study included a total of three studies; a study that was performed in fresh ET cycles,^14^ a study that analyzed FET cycles,^18^ and a study that included both cases.^15^ When a total of 362 patients with adenomyosis who received GnRHa pretreatment for more than 1 month and a control group of 401 patients who did not receive GnRHa pretreatment were compared through meta‐analysis, it was reported that there was no difference in the clinical pregnancy rate. This result is different from the preceding meta‐analysis^2^ that reported positive results of GnRHa pretreatment through two studies.^15^, ^17^ When meta‐analyzing the miscarriage rate through the two studies, there was no difference between the groups.^14^, ^15^ The main limitation is that all studies included are retrospective cohort studies, and their evidence level is relatively low. Also, the high degree of heterogeneity between studies should be considered. (1) The characteristics of the patients included in the studies are diverse. (2) The degree of adenomyosis varies, and characteristics of adenomyosis such as focal or diffuse type should be considered. (3) It should be kept in mind that the results may be different for fresh ET and FET cycles. This is because, in the case of fresh ET, the hormonal profile including estradiol level is changed, which may affect the adverse effects of adenomyosis. In addition, the long‐term use of GnRHa not only increases the gonadotropin dose but also lengthens the stimulation period. (4) Endometriosis, which is often accompanied by adenomyosis, is also a major factor interfering with pregnancy, so its presence should be considered. Randomized control trials (RCTs), which can correct these biases have not yet been carried out, and until now, there is limited evidence to draw a definite recommendation.

In conclusion, hormonal agents including GnRHa treatment are not recommended to increase the spontaneous pregnancy rate in infertile women with adenomyosis. And it is not recommended to administrate GnRHa before fresh embryo transfer cycles to improve IVF outcomes. However, there are studies suggesting that pretreatment with GnRHa may be effective to improve pregnancy rate in FET cycles. Still, there is no RCT, and further studies investigating this possibility are needed in the future.

REFERENCES1

Horton
J
, 
Sterrenburg
M
, 
Lane
S
, 
Maheshwari
A
, 
Li
TC
, 
Cheong
Y
. Reproductive, obstetric, and perinatal outcomes of women with adenomyosis and endometriosis: a systematic review and meta‐analysis. Hum Reprod Update.
2019;25(5):593–633.10.1093/humupd/dmz012313184202

Younes
G
, 
Tulandi
T
. Effects of adenomyosis on in vitro fertilization treatment outcomes: a meta‐analysis. Fertil Steril.
2017;108:483–490.2886554810.1016/j.fertnstert.2017.06.0253

Cozzolino
M
, 
Tartaglia
S
, 
Pellegrini
L
, 
Troiano
G
, 
Rizzo
G
, 
Petraglia
F
. The effect of uterine adenomyosis on IVF outcomes: a systematic review and meta‐analysis. Reprod Sci.
2022;29(11):3177–3193. doi:10.1007/s43032-021-00818-6
349814584

Tanos
V
, 
Lingwood
L
, 
Balami
S
. The importance of the junctional zone of the endometrium in human reproduction. Hum Fertil.
2022;25:4–12.10.1080/14647273.2020.1720316320244095

Shen
M
, 
Liu
X
, 
Zhang
H
, 
Guo
SW
. Transforming growth factor β1 signaling coincides with epithelial‐mesenchymal transition and fibroblast‐to‐myofibroblast transdifferentiation in the development of adenomyosis in mice. Hum Reprod.
2016;31(2):355–369.2668921610.1093/humrep/dev3146

Ota
H
, 
Igarashi
S
, 
Sasaki
M
, 
Tanaka
T
. Distribution of cyclooxygenase‐2 in eutopic and ectopic endometrium in endometriosis and adenomyosis. Hum Reprod.
2001;16(3):561–566.1122822910.1093/humrep/16.3.5617

Bourdon
M
, 
Santulli
P
, 
Jeljeli
M
, 
Vannuccini
S
, 
Marcellin
L
, 
Doridot
L
, et al. Immunological changes associated with adenomyosis: a systematic review. Hum Reprod Update.
2021;27(1):108–129.3309963510.1093/humupd/dmaa0388

Maheshwari
A
, 
Gurunath
S
, 
Fatima
F
, 
Bhattacharya
S
. Adenomyosis and subfertility: a systematic review of prevalence, diagnosis, treatment and fertility outcomes. Hum Reprod Update.
2012;18(4):374–392.2244226110.1093/humupd/dms0069

Khan
KN
, 
Kitajima
M
, 
Hiraki
K
, 
Fujishita
A
, 
Nakashima
M
, 
Ishimaru
T
, et al. Cell proliferation effect of GnRH agonist on pathological lesions of women with endometriosis, adenomyosis and uterine myoma. Hum Reprod.
2010;25(11):2878–2890.2082934310.1093/humrep/deq24010

Khan
KN
, 
Kitajima
M
, 
Hiraki
K
, 
Fujishita
A
, 
Sekine
I
, 
Ishimaru
T
, et al. Changes in tissue inflammation, angiogenesis and apoptosis in endometriosis, adenomyosis and uterine myoma after GnRH agonist therapy. Hum Reprod.
2010;25(3):642–653.2000888810.1093/humrep/dep43711

Xie
M
, 
Yu
H
, 
Zhang
X
, 
Wang
W
, 
Ren
Y
. Elasticity of adenomyosis is increased after GnRHa therapy and is associated with spontaneous pregnancy in infertile patients. J Gynecol Obstet Hum Reprod.
2019;48(10):849–853.3106749810.1016/j.jogoh.2019.05.00312

Hou
X
, 
Xing
J
, 
Shan
H
, 
Mei
J
, 
Sun
Y
, 
Yan
G
, et al. The effect of adenomyosis on IVF after long or ultra‐long GnRH agonist treatment. Reprod BioMed Online.
2020;41(5):845–853.3297287310.1016/j.rbmo.2020.07.02713

Lan
J
, 
Wu
Y
, 
Wu
Z
, 
Wu
Y
, 
Yang
R
, 
Liu
Y
, et al. Ultra‐long GnRH agonist protocol during IVF/ICSI improves pregnancy outcomes in women with adenomyosis: a retrospective cohort study. Front Endocrinol.
2021;12:609771.10.3389/fendo.2021.609771PMC82020823413585814

Chen
M
, 
Luo
L
, 
Wang
Q
, 
Gao
J
, 
Chen
Y
, et al. Impact of gonadotropin‐releasing hormone agonist pre‐treatment on the cumulative live birth rate in infertile women with adenomyosis treated with IVF/ICSI: a retrospective cohort study. Front Endocrinol. 2020;11:318.10.3389/fendo.2020.00318PMC72738423254749015

Park
CW
, 
Choi
MH
, 
Yang
KM
, 
Song
IO
. Pregnancy rate in women with adenomyosis undergoing fresh or frozen embryo transfer cycles following gonadotropin‐releasing hormone agonist treatment. Clin Exp Reprod Med. 2016;43(3):169–173.2768904010.5653/cerm.2016.43.3.169PMC503931016

Wu
Y
, 
Huang
J
, 
Zhong
G
, 
Lan
J
, 
Lin
H
, 
Zhang
Q
, et al. Long‐term GnRH agonist pretreatment before frozen embryo transfer improves pregnancy outcomes in women with adenomyosis. Reprod Biomed Online. 2022;44(2):380–388.3489582710.1016/j.rbmo.2021.10.01417

Niu
Z
, 
Chen
Q
, 
Sun
Y
, 
Feng
Y
. Long‐term pituitary downregulation before frozen embryo transfer could improve pregnancy outcomes in women with adenomyosis. Gynecol Endocrinol.
2013;29(12):1026–1030.2400690610.3109/09513590.2013.82496018

Li
M
, 
Xu
L
, 
Zhao
H
, 
Du
Y
, 
Yan
L
. Effects of artificial cycles with and without gonadotropin‐releasing hormone agonist pre‐treatment on frozen embryo transfer outcomes in patients with adenomyosis. Sci Rep.
2021;11(1):19326.3458857610.1038/s41598-021-98918-5PMC8481533

## 8 CQ7: DOES ADENOMYOSIS INFLUENCE THE PREGNANCY COMPLICATION?

1. Adenomyosis has a detrimental effect on pregnancy outcomes. This includes a higher risk for miscarriage, preterm birth, small for gestational age, pregnancy‐induced hypertension, Caesarean section, fetal malpresentation, and postpartum hemorrhage.

Level of Evidence: 1b

Grade of recommendation: A

2. Adenomyosis is associated with a lower clinical pregnancy rate and higher miscarriage rate, even with ART.

Level of Evidence: 1b

Grade of recommendation: A

3. An ultra‐long GnRHa protocol may increase fertility rates with ART for patients with adenomyosis.

Level of Evidence: 2a

Grade of recommendation: B

4. Pregnant women with adenomyosis should be closely monitored for pregnancy‐related complications.

Level of Evidence: 1b

Grade of recommendation: A

Meta‐analyses, systematic Reviews 3

Case studies: Prospective 1, retrospective 1

### 8.1 Commentary

Several meta‐analyses have been performed on the impact of adenomyosis on pregnancy outcomes. To date, there have been no randomized controlled studies and all studies analyzed were either prospective case‐control or retrospective cohort studies^1^, ^2^, ^3^

This means there is much heterogeneity among the studies with variation in the method of diagnosis/ classification/severity of adenomyosis, maternal age, parity, coexistence of endometriosis, and previous medical history. Method of conception is also a confounding factor as it is known that ART is an independent risk factor for pregnancy complications.

One of the largest meta‐analyses by Horton et al.^1^ included 100 eligible studies for both endometriosis and adenomyosis. Meta‐analysis demonstrated that adenomyosis is associated with reduced clinical pregnancy rates (OR 0.57, CI 0.43–0.76, *p* < 0.001; *n* = 7), live birth rate (LBR) was reduced (OR 0.45, CI 0.24–0.86, *p* = 0.02; *n* = 5), and there was an increased risk of miscarriage (OR 3.49, CI 1.41–8.65, *p* = 0.007; *n* = 6). There was also increased risk for preterm delivery (PTD) (OR 2.74, CI 1.89–3.97, *p* < 0.001; *n* =5), small for gestational age fetus (SGA) (OR 3.90, CI 2.10–7.25, *p* < 0.001; *n* = 2), lower segment cesarean section (LSCS) (OR 2.62, CI 1.00–6.89, *p* = 0.05; *n* = 3), and pre‐eclampsia (PET) (OR 7.87, CI 1.26–49.20, *p* = 0.03; *n* = 2).

Another more recent meta‐analysis by Nirgianakis et al.^2^ considered sensitivity analysis of maternal age and the coexistence of endometriosis. After matching for age and endometriosis, they demonstrated that adenomyosis is associated with a higher risk for miscarriage (OR 2.50; 95%CI 1.26–4.95; *n* = 6), PTD (OR 2.83; 95%CI 2.18–3.69; *n* = 5), PET (OR 4.32; 95% CI 1.68–11.09; *n* = 4), Caesarean section (OR 4.44; 95% CI 2.64–7.47; *n* = 2), fetal malpresentation (OR 3.05; 95% CI 1.60–5.81; *n* = 2), postpartum hemorrhage (PPH) (OR 2.90; 95% CI 1.39–6.05; *n* = 3), SGA (OR 2.10; 95% CI 1.17–3.77; *n* = 2), and low birthweight (LBW) (OR 2.82; 95% CI 1.20–6.62; *n* = 3).

When different subgroups of ART protocols were analyzed, Nirgianakis et al.^2^ found that patients who were under ultra‐long or modified ultra‐long protocols performed no different in terms of clinical pregnancy rates (OR 0.78, 95% CI 0.45–1.35; *n* = 3), LBRs (OR 0.64; 95% CI 0.19–2.14; *n* = 2) or miscarriage rates (OR 1.23; 95% CI 0.31–4.91; *n*= 3) compared to controls (no adenomyosis). They postulated that ultra‐long protocols may produce a period of estrogen deficiency that may temporarily inactivate adenomyosis, reduce uterine volume and normalize some of the distorted endometrial functions, with an improvement in fertility outcomes. It is, therefore, plausible that prolonged GnRHa may be administered before endometrial preparation for vitrified embryos, to improve fertility outcomes.

However, in a retrospective study comparing no pretreatment, medication, and surgery for adenomyosis, Tamura et al.^4^ found no difference in pregnancy complication rates among the 3 groups. A further study by Zhang et al.^5^ using a long downregulation protocol for in vitro fertilization in a retrospective case‐control series found that adenomyosis had a lower implantation rate, clinical pregnancy rate, LBR, and a higher spontaneous miscarriage rate than endometriosis controls. The difference in the results of studies could be explained by the heterogeneity of the treatment protocol, among other factors.

Both meta‐analysis^2^ and retrospective case study^4^ did not show any difference in fertility outcomes between focal and diffuse adenomyosis in terms of miscarriage, premature labor, and LBR. Tamura et al., however, did find a higher miscarriage rate and cervical incompetency in patients with adenomyosis measuring >60 mm.^4^

REFERENCES1

Horton
J
, 
Sterrenburg
M
, 
Lane
S
, 
Maheshwari
A
, 
Li
TC
, 
Cheong
Y
. Reproductive, obstetric, and perinatal outcomes of women with adenomyosis and endometriosis: a systematic review and meta‐analysis. Human Reprod Update. 2019;25:592–632.10.1093/humupd/dmz012313184202

Nirgianakis
K
, 
Kalaitzopoulos
DR
, 
Schwartz
ASK
, 
Spaanderman
M
, 
Kramer
BW
, 
Mueller
MD
, et al. Fertility, pregnancy and neonatal outcomes of patients with adenomyosis: a systematic review and meta‐analysis. Reprod Biomed Online. 2021;42(1):185–206.3319113110.1016/j.rbmo.2020.09.0233

Razavi
M
, 
Maleki‐Hajiagha
A
, 
Sepidarkish
M
, 
Rouholamin
S
, 
Almasi‐Hashiani
A
, 
Rezaeinejad
M
. Systematic review and meta‐analysis of adverse pregnancy outcomes after uterine adenomyosis. Int J Gynaecol Obstet.
2019;145(2):149–157.3082880810.1002/ijgo.127994

Tamura
H
, 
Kishi
H
, 
Kitade
M
, 
Asai‐Sato
M
, 
Tanaka
A
, 
Murakami
T
, et al. Complications and outcomes of pregnant women with adenomyosis in Japan. Reprod Med Biol.
2017;16(4):330–336.2925948610.1002/rmb2.12050PMC57158915

Zhang
J
, 
Hu
L
, 
Bu
Z
, 
Sun
Y
. Impact of uterine adenomyosis on pregnancy outcomes in women undergoing in vitro fertilization treated with a long‐term pituitary downregulation protocol. Front Endocrinol (Lausanne).
2021;12:655803.3448986110.3389/fendo.2021.655803PMC8416510

## 9 CQ8: DOES THE REMOVAL OF ADENOMYOSIS LESIONS INFLUENCE PREGNANCY COMPLICATIONS?

Women may be offered adenomyomectomy to improve pregnancy outcomes and should be counseled regarding complications of the surgery, such as uterine rupture and placenta accrete.

Level of evidence: 4

Grade of recommendation C

Observational study: Prospective 3, Retrospective 4

Case series: Retrospective 4

### 9.1 Commentary

Adenomyomectomy may improve pregnancy outcomes.^1^ Lesion debulking improves other outcomes such as bleeding and pain and may increase the chances of conceiving. The patient should also be counseled regarding complications of the surgery, albeit rare, and this may include uterine rupture and placental accreta. Adenomyotic foci often deeply invade the myometrium making the complete resection of the lesions almost impossible and the removal of healthy myometrial tissue almost inevitable, which leads to poor healing of the scar, increasing the risk of uterine rupture during pregnancy.^2^

Although only 10 pregnant cases after adenomyomectomy, 3 patients resulted in preterm delivery and had a very thin uterus to the extent that the fetus could be observed through the uterine wall. A shortened cervical length should be paid special attention to in pregnant women after adenomyomectomy.^3^ Twenty‐two patients were evaluated to monitor pregnancy and delivery outcomes after the adenomyomectomy. Placental abnormality was found in the 4 cases, which included 2 placental accreta and 2 previa. One case of uterine rupture during pregnancy (4.5%, 1/22) at 27 weeks of gestation.^4^

In terms of the adenomyomectomy, the method in which adenomyotic tissues are radically excised and the uterine wall is reconstructed by a triple‐flap without overlapping sutures shown to prevent uterine rupture in subsequent pregnancies. Of 26 women who wished to conceive, 16 became pregnant, 14 (53.8%) went to term and delivered a healthy baby and there were no cases of uterine rupture.^5^ The wall thickness of the excised uterus was highly associated with uterine rupture. In pregnant women who underwent uterine‐sparing surgery for diffuse‐type adenomyosis, optimum wall thickness for conception and preventing uterine rupture during pregnancy may range from 9 to 15 mm.^6^

In Japan, a nationwide survey for 5 years was performed to clarify the frequency of occurrence of uterine rupture and its prognosis. Seven uterine rupture cases after adenomyomectomy were reported (Median; abdominal: 30.0 weeks, laparoscopic: 32.0 weeks of pregnancy). The neonatal prognosis was poorer in cases of pregnancy after adenomyomectomy in comparison with postcesarean section cases.^7^

REFERENCES1

Saremi
A
, 
Bahrami
H
, 
Salehian
P
, 
Hakak
N
, 
Pooladi
A
. Treatment of adenomyomectomy in women with severe uterine adenomyosis using a novel technique. Reproduct Biomed Online. 2014;28(6):753–760.10.1016/j.rbmo.2014.02.008247685582

Kishi
Y
, 
Yabuta
M
, 
Taniguchi
F
. Who will benefit from uterus‐sparing surgery in adenomyosis‐associated subfertility?
Fertil Steril. 2014;102(3):802–807.2495477410.1016/j.fertnstert.2014.05.0283

Sugiyama
M
, 
Takahashi
H
, 
Baba
Y
, 
Taneichi
A
, 
Suzuki
H
, 
Usui
R
, et al. Perinatal outcome of pregnancy after adenomyomectomy: summary of 10 cases with a brief literature review. J Matern Fetal Neonatal Med. 2020;33(24):4145–4149.3088999910.1080/14767058.2019.15978454

Kwack
JY
, 
Lee
SJ
, 
Kwon
YS
. Pregnancy and delivery outcomes in the women who have received adenomyomectomy: performed by a single surgeon by a uniform surgical technique. Taiwan J Obstet Gynecol. 2021;60(1):99–102.3349501810.1016/j.tjog.2020.11.0155

Osada
H
, 
Silber
S
, 
Kakinuma
T
, 
Nagaishi
M
, 
Kato
K
, 
Kato
O
. Surgical procedure to conserve the uterus for future pregnancy in patients suffering from massive adenomyosis. Reprod Biomed Online. 2011;22(1):94–99.2111875110.1016/j.rbmo.2010.09.0146

Otsubo
Y
, 
Nishida
M
, 
Arai
Y
, 
Ichikawa
R
, 
Taneichi
A
, 
Sakanaka
M
. Association of uterine wall thickness with pregnancy outcome following uterine‐sparing surgery for diffuse uterine adenomyosis. Aust N Z J Obstet Gynaecol. 2016;56(1):88–91.2651593610.1111/ajo.124197

Makino
S
, 
Takeda
S
, 
Kondoh
E
, 
Kawai
K
, 
Takeda
J
, 
Matsubara
S
, et al. National survey of uterine rupture in Japan: Annual report of Perinatology Committee, Japan Society of Obstetrics and Gynecology, 2018. J Obstet Gynaecol Res. 2019;45(4):763–765.3085472510.1111/jog.13924

## 10 CQ 9: DOES HORMONAL TREATMENT OF ADENOMYOSIS INFLUENCE THE PREGNANCY OUTCOME?

1. Hormonal treatments improve the pregnancy rate in ART.

Level of Evidence: 3b

Grade of Recommendation: C

2. Hormonal treatments influence the neonatal outcome in ART.

Level of Evidence: 4

Grade of Recommendation: C

3. Hormonal treatments influence the miscarriage rate in ART.

Level of Evidence: 4

Grade of Recommendation: C

4. Hormonal treatments influence the pregnancy rate in natural conception.

Level of Evidence: 4

Grade of Recommendation: C

Cohorts 9

Case Reports 3

### 10.1 Commentary

Most of the gathered studies to answer this clinical question were cohorts and case reports, without any meta‐analysis discussing the pregnancy complications and neonatal outcomes derived from the hormonal treatment of adenomyosis. The hormonal treatment whose pregnancy complications and neonatal outcomes had been extensively studied was a gonadotropin‐releasing hormone agonist (GnRHa). The rationale for using the GnRHa is to capitalize the negative feedback effect of GnRH elevation, reducing the secretion of FSH and LH from the pituitary hence creating the hypoestrogenic environment and suppressing the endometrial cell proliferation.^1^ Aside from the systemic and local hypoestrogenic effect, the GnRHa has a direct antiproliferative effect within the myometrium through the action on the GnRH receptors expressed by adenomyotic lesions. The reduced expression of nitric oxide synthases, peroxynitrite, and serum levels of nitrite/nitrate are found in the GnRHa treatment, which is usually increased in adenomyosis.^2^ It is also thought that GnRHa may suppress ovulation and the production of estrogen, decrease the expression of aromatase cytochrome P450 in the eutopic endometrium, and reduce the inflammatory reaction and angiogenic response in endometrial tissues.^1^

The regimen of GnRHa has been investigated as a sole agent therapy, as an adjunctive treatment to surgical management, and as an endometrial preparation for assisted reproductive technology (ART). GnRHa administration alone has been studied in several case reports, with the length of administration ranging from 3 months to 3 years. The case reports showed successful natural conception with a duration of up to 6 months after the completed GnRHa treatment.^3^, ^4^, ^5^, ^6^ Several cohorts also showed the combination of GnRHa with adenomyomectomy had a statistical difference in clinical pregnancy rate compared to GnRHa alone (OR 0.25, 95% CI 0.09–0.68, *I* ^2^ = 0%).^7^, ^8^ However, one study showed the GnRHa intervention only did not have a significant difference in miscarriage rate (OR 0.3810, 95% CI 0.0317–4.810, *p* = 0.4469) and ectopic pregnancy (OR 2.5814, 95% CI 0.0991–67.2711, *p* = 0.5686) compared to the addition of adenomyomectomy.^7^

Several cohorts have supported the evidence of GnRHa as a part of endometrial preparation for the ART protocol. It was hypothesized that GnRHa restores the endometrial receptivity in women with adenomyosis, as simulated from the animal study, hence the improved implantation and pregnancy rate in the clinical studies. With the improvement of endometrial receptivity assays such as homeobox genes, leukemia inhibitory factor, and pinopodes, the adjunctive GnRHa is widely considered in the ART protocol.^9^ The GnRH agonist whose role is to induce the hypothalamic‐pituitary‐gonadal‐axis is also often accompanied by the intake of 20–60 mg/day progesterone as a luteal support scheme after the embryo transfer and administration of human chorionic gonadotropin (hCG) to induce the maturation of oocyte.^10^, ^11^, ^12^, ^13^, ^14^, ^15^ In the in vitro fertilization (IVF) studies, GnRHa statistically improved the biochemical pregnancy (OR 3.31, 95% CI 2.28–4.80, *I* ^2^ = 93%)^13^, ^14^, ^15^, ^16^, ^17^ and ongoing pregnancy (OR 2.52, 95% CI 1.87–3.40, *I* ^2^ = 30%).^12^, ^13^ However, there are no statistical differences in a clinical pregnancy (OR 1.13, 95% CI 0.97–1.31, *I* ^2^ = 90%),^13^, ^14^, ^15^, ^16^, ^17^ miscarriage rate (OR 0.75, 95% CI 0.52–1.10, *I* ^2^ = 23%),^10^, ^11^, ^12^, ^14^, ^15^ preterm labor (OR 1.28, 95% CI 0.55–2.98, *I* ^2^ = 0%),^11^, ^12^, ^13^, ^14^ and live birth rate (OR 1.22, 95% CI 0.95–1.58, *I* ^2^ = 87%) between GnRHa and non‐GnRHa treatment.^10^, ^11^, ^12^, ^14^, ^16^

The usage of GnRHa in IVF studies varied from one study to another. Some studies used an ultra‐long GnRHa protocol (triptorelin, leuprorelin, or diphereline 3.75 mg intramuscularly, every 28 days for at least 1–3 months before ovarian stimulation).^10^, ^11^, ^12^, ^13^, ^14^, ^15^ Others used a long GnRH agonist (triptorelin 0.1 mg/day for 10 days followed by 0.05 mg/day until the day of hCG injection) or short‐term GnRHa (Buserelin acetate, 50 IU subcutaneously up to 4 days).^6^, ^10^ Ultra‐long GnRH protocol served as a protective factor for clinical pregnancy rate (OR 1.925, 95% CI 1.137–3.250, *p* = 0.015), implantation rate (OR 1.694, 95% CI 1.006–2.854, *p* = 0.047) and live birth rate (OR 1.704, 95% CI 1.012–2.859, *p* = 0.04) compared to long GnRHa protocol.^10^ However, there were no significant differences in miscarriage rate (OR 0.811, 95% CI 0.39–1.78, *p* = 0.39) between these two GnRHa protocol.^10^ The administered ultra‐long GnRH protocol showed a statistically higher pregnancy rate in women with diffuse adenomyosis in several cohorts. The focal adenomyosis did not exhibit a statistical difference hence the usage of long GnRH protocol was more preferred in terms of time consumption and cost.^10^, ^11^, ^12^, ^13^, ^14^, ^15^, ^16^, ^17^

The preference for embryo transfers also contributed to the adjunctive GnRH agonist treatment to ART. One cohort study showed that a combination of GnRH agonist and frozen embryo transfer had a significant protective factor for the implantation rate, clinical pregnancy rate, live birth rate, and miscarriage rate compared to a fresh embryo transfer.^16^

The pregnancy complications and neonatal outcome from other hormonal treatments of Adenomyosis such as progestins, oral contraceptives (OCs), danazol, selective estrogen receptor modulators (SERMs), selective progesterone receptor modulators (SPRMs), or aromatase inhibitors (AIs) have yet to be studied. The objectives of these agents were to inhibit ovulation, abolition of menstruation, and achieve a stable steroid hormone milieu. These agents act mainly based on the hypothalamic‐pituitary‐gonadal‐axis principle, which plays a pivotal role in mammalian reproduction. Danazol is thought to create a hyperandrogenic environment, whereas OCs and progestins create a hyperprogestogenic environment.^1^, ^2^ Further investigation concerning the pregnancy complications and neonatal outcomes of these hormonal treatments needs to be conducted.

REFERENCES1

Tsui
KH
, 
Lee
WL
, 
Chen
CY
, 
Sheu
BC
, 
Yen
MS
, 
Chang
TC
, et al. Medical treatment for adenomyosis and/or adenomyoma. Taiwanese J Obstet Gynecol.
2014;53(4):459–465. 10.1016/j.tjog.2014.04.024
255106832

Vannuccini
S
, 
Luisi
S
, 
Tosti
C
, 
Sorbi
F
, 
Petraglia
F
. Role of medical therapy in the management of uterine adenomyosis. Fertil Steril.
2018;109(3):398–405. 10.1016/j.fertnstert.2018.01.013
295668523

Harada
T
, 
Mon Khine
Y
, 
Kaponis
A
, 
Nikellis
T
, 
Decavalas
G
, 
Taniguchi
F
. The impact of adenomyosis on women's fertility. Obstet Gynecol Surv.
2016;71(9):557–568. 10.1097/OGX.0000000000000346
27640610PMC50499764

Nelson
JR
, 
Corson
SL
. Long‐term management of adenomyosis with a gonadotropin‐releasing hormone agonist: a case report. Fertil Steril.
1993;59(2):441–443. 10.1016/s0015-0282(16)55704-5
84256435

Silva
PD
, 
Perkins
HE
, 
Schauberger
CW
. Live birth after treatment of severe adenomyosis with a gonadotropin‐releasing hormone agonist. Fertil Steril.
1994;61(1):171–172. 10.1016/s0015-0282(16)56471-1
82938326

Huang
FJ
, 
Kung
FT
, 
Chang
SY
, 
Hsu
TY
. Effects of short‐course buserelin therapy on adenomyosis. A report of two cases. J Reprod Med.
1999;44(8):741–744.104835487

al Jama
FE
. Management of adenomyosis in subfertile women and pregnancy outcome. Oman Med J.
2011;2(3):178–181. 10.5001/omj.2011.43
PMC3191687220434118

Wang
PH
, 
Fuh
JL
, 
Chao
HT
, 
Liu
WM
, 
Cheng
MH
, 
Chao
KC
. Is the surgical approach beneficial to subfertile women with symptomatic extensive adenomyosis?
J Obstet Gynaecol Res.
2009;35(3):495–502. 10.1111/j.1447-0756.2008.00951.x
195273899

Guo
S
, 
Li
Z
, 
Yan
L
, 
Sun
Y
, 
Feng
Y
. GnRH agonist improves pregnancy outcome in mice with induced adenomyosis by restoring endometrial receptivity. Drug Des Devel Ther.
2018;12:1621–1631. 10.2147/DDDT.S162541
PMC59952912992203710

Hou
X
, 
Xing
J
, 
Shan
H
, 
Mei
J
, 
Sun
Y
, 
Yan
G
, et al. The effect of adenomyosis on IVF after long or ultra‐long GnRH agonist treatment. Reprod Biomed Online.
2020;41(5):845–853. 10.1016/j.rbmo.2020.07.027
3297287311

Li
M
, 
Xu
L
, 
Zhao
H
, 
Du
Y
, 
Yan
L
. Effects of artificial cycles with and without gonadotropin‐releasing hormone agonist pretreatment on frozen embryo transfer outcomes in patients with adenomyosis. Sci Rep.
2021;11(1):19326. 10.1038/s41598-021-98918-5
34588576PMC848153312

Pan
D
, 
Yang
J
, 
Zhang
N
, 
Wang
L
, 
Li
N
, 
Shi
J
, et al. Gonadotropin‐releasing hormone agonist downregulation combined with hormone replacement therapy improves the reproductive outcome in frozen‐thawed embryo transfer cycles for patients of advanced reproductive age with idiopathic recurrent implantation failure. Reprod Biol Endocrinol. 2022;20(1):26. 10.1186/s12958-022-00897-3
35115007PMC881217913

Niu
Z
, 
Chen
Q
, 
Sun
Y
, 
Feng
Y
. Long‐term pituitary downregulation before frozen embryo transfer could improve pregnancy outcomes in women with adenomyosis. Gynecol Endocrinol. 2013;29(12):1026–1030. 10.3109/09513590.2013.824960
2400690614

Chen
M
, 
Luo
L
, 
Wang
Q
, 
Gao
J
, 
Chen
Y
, 
Zhang
Y
, et al. Impact of gonadotropin‐releasing hormone agonist pre‐treatment on the cumulative live birth rate in infertile women with adenomyosis treated with IVF/ICSI: a retrospective cohort study. Front Endocrinol (Lausanne). 2020;11:318. 10.3389/fendo.2020.00318
32547490PMC727384215

Kolanska
K
, 
Cohen
J
, 
Bendifallah
S
, 
Selleret
L
, 
Antoine
JM
, 
Chabbert‐Buffet
N
, et al. Pregnancy outcomes after controlled ovarian hyperstimulation in women with endometriosis‐associated infertility: GnRH‐agonist versus GnRH‐antagonist. J Gynecol Obstet Hum Reprod. 2017;46(9):681–686. 10.1016/j.jogoh.2017.09.007
2897013516

Wu
Y
, 
Huang
J
, 
Zhong
G
, 
Lan
J
, 
Lin
H
, 
Zhang
Q
. Long‐term GnRH agonist pretreatment before frozen embryo transfer improves pregnancy outcomes in women with adenomyosis. Reprod Biomed Online. 2022;44(2):380–388. 10.1016/j.rbmo.2021.10.014
3489582717

Lan
J
, 
Wu
Y
, 
Wu
Z
, 
Wu
Y
, 
Yang
R
, 
Liu
Y
, et al. Ultra‐long GnRH agonist protocol during IVF/ICSI improves pregnancy outcomes in women with adenomyosis: a retrospective cohort study. Front Endocrinol (Lausanne). 2021;12:609771. 10.3389/fendo.2021.609771
34135858PMC8202082

## CONFLICT OF INTEREST STATEMENT

Tasuku Harada is an Editorial Board member of Reproductive Medicine and Biology and a 1st author of this article. To minimize bias, he was excluded from all editorial decision‐making related to the acceptance of this article for publication. Other coauthors declare no conflicts of interest for this article.
